# Endostructural and periosteal growth of the human humerus

**DOI:** 10.1002/ar.25048

**Published:** 2022-08-25

**Authors:** Thomas George O'Mahoney, Tristan Lowe, Andrew Timothy Chamberlain, William Irvin Sellers

**Affiliations:** ^1^ School of Life Sciences Anglia Ruskin University Cambridge UK; ^2^ School of Earth and Environmental Sciences University of Manchester Manchester UK; ^3^ Henry Moseley X‐Ray Imaging Facility University of Manchester Manchester UK

**Keywords:** biomechanics, geometric morphometrics, humerus, ontogeny

## Abstract

The growth and development of long bones are of considerable interests in the fields of comparative anatomy and palaeoanthropology, as evolutionary changes and adaptations to specific physical activity patterns are expected to be revealed during bone ontogeny. Traditionally, the cross‐sectional geometry of long bones has been examined at discrete locations usually placed at set intervals or fixed percentage distances along the midline axis of the bone shaft. More recently, the technique of morphometric mapping has enabled the continuous analysis of shape variation along the shaft. Here we extend this technique to the full sequence of late fetal and postnatal development of the humeral shaft in a modern human population sample, with the aim of establishing the shape changes during growth and their relationship with the development of the arm musculature and activity patterns. A sample of modern human humeri from individuals of age ranging from 24 weeks in utero to 18 years was imaged using microtomography at multiple resolutions and custom Matlab scripts. Standard biomechanical properties, cortical thickness, surface curvature, and pseudo‐landmarks were extracted along radial vectors spaced at intervals of 1° at each 0.5% longitudinal increment measured along the shaft axis. Heat maps were also generated for cortical thickness and surface curvature. The results demonstrate that a whole bone approach to analysis of cross‐sectional geometry is more desirable where possible, as there is a continuous pattern of variation along the shaft. It is also possible to discriminate very young individuals and adolescents from other groups by relative cortical thickness, and also by periosteal surface curvature.

## 1 INTRODUCTION

This article sets out to understand the fashion in which localized cortical thickness, biomechanical resistance to torsional stress, localized surface curvature and overall diaphyseal curvature vary throughout ontogeny and, the degree to which these factors co‐vary (or not). As such, in this section, firstly we briefly summarize the state of knowledge of ontogeny of long bones, and specifically the human humerus. We then focus on the previous work on juvenile long‐bone biomechanics, including cross‐sectional geometry and diaphyseal curvature. Finally, we look at the advantages of virtual morphometric methods such as geometric morphometrics (GMM) and “morphometric mapping” (Zollikofer & Ponce de León, 2001) and how these can be of specific use for the analysis of external morphology and endostructural variation of long bones throughout growth.

### 1.1 Why the humerus?

The humerus is a major long bone in the human body that after the acquisition of bipedality is not involved in habitual locomotion. As such, differences between groups can often be larger than that in the bones associated with walking (e.g., the tibia and femur) (e.g., Churchill, 1996; De Groote, 2011a, 2011b; Pearson et al., 2006; Ruff, 2005a, 2005b) as the humerus is not strictly a weight‐bearing bone but potentially responds to a diverse range of biomechanical stresses generated by upper limb usage. It grows in the same manner as other long bones, however, with the shaft being the major portion that grows (Gray & Gardner, 1969). Approximately, 80% of humerus growth occurs at the proximal growth plate (Pritchett, 1991), as can be seen by the distinctive shape the distal portion assumes even at very early embryonic stages. Prior to birth, it is assumed that much of the form of the humerus is genetically pre‐programmed, as the forces it withstand are not as high in utero as those experienced by the tibia and femur (which withstands greater forces due to the kicking reflex) (Verbruggen et al., 2016, 2018). Aspects of the development of this bone appear to be linked to expression of two genes, Collagen X and Indian hedgehog, which work in tandem with biophysical stimuli of embryonic muscle contractions (Nowlan et al., 2008) and knockout mouse models suggest that the development of musculature in this region has an important factor to play in the normal development of the humerus (Nowlan et al., 2010). Examination of individuals who have continued with normal postnatal musculoskeletal development in this region allows us to understand possible developmental pathways. It can also allow a more nuanced understanding of how pathological conditions may manifest themselves than a simple visual analysis will.

### 1.2 Some previous developmental studies of long bone size and shape

There is debate as to how much of the internal structure of the long bones is dictated by either genetics or behavior. Long bones are often modeled by morphologists as straight cylinders so that their key functional properties such as resistance to bending, torsion, and so forth can be assessed by means of simple analyses of cross‐sectional shape. Unfortunately, not all long bones are straight cylinders, and in fact, correction for curvature can alter estimates of bone stiffness by up to 15% (Brassey et al., 2013).

Much of the work on cross‐sectional geometry of juvenile long bones has built on a range of studies including analyses of adult hominin and primate skeletal material (e.g., Churchill, 1996; Davies & Stock, 2014; Niinimäki et al., 2013; Rhodes & Knüsel, 2005; Ruff & Trinkaus, 2012; Shaw et al., 2014; Trinkaus et al., 1994) as well as experimental work in non‐primates (e.g., Lieberman et al., 2001, 2003) and in living humans (e.g., Nikander et al., 2006; Shaw & Stock, 2009a, 2009b).

To understand the functional significance and also the variation of adult long bone morphology, it is important to ascertain the stages within the growth trajectory at which morphological change occurs and their significance (Cowgill, 2010; Gosman et al., 2013; Ruff, 2005a, 2005b; Smith & Buschang, 2004). To this end, several studies have looked at growth of human long bones using different techniques, namely: histology (Cambra‐Moo, 2014; Kember & Sissons, 1976; Maggiano et al., 2016); radiography (Tanner, 1981); intermembral indices and/or cross‐sectional geometry (e.g., Cowgill, 2010; Gosman et al., 2013; Harrington, 2010; Kondo & Dodo, 2002a, 2002b; Osipov et al., 2016; Ruff, 2003a, 2003b; Ruff et al., 1994; Ruff et al., 2002; Trinkaus et al., 2002; Zilhão & Trinkaus, 2002); GMM (Frelat & Mitteroecker, 2011); and analysis of torsion (Cowgill, 2007).

In very early development, normal stresses will enable the genetically programmed process of bone formation to occur, but *abnormal stresses*, either caused by external factors (e.g., pathogens attacking the mother, severe malnutrition of mother, and trauma) or internal genetic factors such as deleterious mutations, will interfere with this program of bone formation. To this end, it is important in baseline studies to try and study nonpathological material for the creation of reference sequences. Cowgill (2010) was able to demonstrate that observable differences in population‐level cross‐sectional properties at the 50% of maximum length (hereafter referred to as increment) of both the humerus and femur existed very early, often before 1 year of age. This suggests that a complex long‐term interplay between population genetics and environment influences both long bone robusticity and bone shape.

It is apparent, however, that analysis of just the cross‐section of the humerus at the 50% increment may miss out variation and patterns that may be biomechanically meaningful. To this end, it is increasingly common for studies to analyze several locations throughout the diaphysis (Churchill, 1996; Davies & Stock, 2014; Niinimäki et al., 2013; Rhodes & Knüsel, 2005; Shaw et al., 2014). However, with decreasing cost and increasing resolution of imaging modalities (which broadly follow an equivalent of “Moore's Law”), it is increasingly desirable to attempt a holistic “whole bone” approach. Davies and Stock (2014) examined the periosteal contours of adult long bones from laser scans in order to extract cross‐sectional properties, examining 1% longitudinal increments. Shaw et al. (2014) examined 5% increments of the femur to examine sex differences in cortical structure. Morimoto et al. (2011) analyzed the entire diaphysis of femora from chimpanzees ranging from infant to adult stages using a technique previously dubbed “morphometric mapping” (Zollikofer & Ponce de León, 2001). This technique borrows broadly from the techniques used in functional brain imaging and enables the quantification of surfaces that are relatively landmark free, such as immature long bone diaphyses. This technique, using a different statistical treatment, was also used by Puymerail (2013) (and also Puymerail, Ruff, et al., 2012, Puymerail, Volpato, et al., 2012, Puymerail et al., 2013; Ruff et al., 2015) in the exploration of the differences between the adult femur of *Homo sapiens*, *Homo neanderthalensis*, *Homo erectus*, and *Pan troglodytes*.

In this study, using automated extraction of percentage cortical area, second moment of area, and maximum/minimum second moments of area (*I*_max_/*I*_min_), we aim to bring more fine‐grained data to bear on the question of structural differentiation of areas of the humerus throughout growth.

Development of humeral curvature has recently been studied by Hambücken (2012). Here, she looked at the curvature of the humerus in a medieval cemetery sample in the medial view throughout development and concluded that the humerus tends to start development as a relatively straight bone, and after the age of 1 year, a convex posterior curvature manifests itself. This tends to persist to around 12 years of age. At the age of around three and a half years, a distal curvature can be observed as well; however, a definite curvature is not fully apparent until skeletal maturity is reached (Hambücken, 2012). More work on further samples is needed to establish whether these observations can be generalized to multiple populations. This article will approach this through a GMM workflow, which although yielding slightly different results, should be complementary in interpretation.

### 1.3 Virtual morphometric approaches

#### 1.3.1 GMM approaches

The suite of statistical techniques associated with GMM has proved to be extremely useful in distinguishing group affiliation in multiple species and multiple anatomical regions (see e.g., Adams & Otarola‐Castillo, 2013). The adoption of GMM analysis in the study of the growth of juvenile long bones has, however, been less common, as much of young long bones lack recognizable Type 1 or Type 2 landmarks. Authors including Morimoto et al. (2011) have proposed the extraction of “pseudo‐landmarks” at predefined intervals to try and overcome this lack. This is especially appropriate to the study of diaphyseal morphology here, as it would mean that the morphology is easily subjected to standardized GMM workflows post extraction of the pseudo‐landmarks. Morimoto et al. (2011) applied this workflow to the diaphysis of juvenile chimpanzee femora and demonstrated that this approach yielded very satisfactory results. We aim to further demonstrate the utility of this approach in the extraction and analysis of pseudo‐landmark data in our samples (detailed below).

#### 1.3.2 Landmark free approaches

Where properties such as localized cortical thickness or surface curvature, are of interest, the approach dubbed “morphometric mapping” has been suggested (Zollikofer & Ponce de León, 2001). This technique borrows inspiration from functional brain imaging, where localized differences in thickness (in our case, cortical thickness) are highlighted on a three‐dimensional model through colorization using a scaled “heatmap.” As the diaphysis of a long bone is broadly cylindrical in form, the heat map can be “unzipped” and “unrolled” (Bondioli et al., 2010; Morimoto et al., 2011; Zollikofer & Ponce de León, 2001) and projected to a two‐dimensional (2D) graphical representation (the “map”) with relatively little distortion in the humerus. Here we look at two characteristics: cortical thickness and radial curvature of the periosteum.

Cortical thickness is measured radially at each slice from the per‐slice centroid of each cross section of the diaphysis, following Jashashvili et al. (2015). The technique of Bondioli et al. (2010) made measurements from the linear medial axis, which can introduce distortions in thickness measurements (Dupej et al., 2017). The use of simple linear rays for this measurement (rather than tangents of the internal points used by Morimoto et al., 2011) is appropriate for the humerus as the cross section is approaching circular. It also reduces computational complexity. The code describing this is available in Supplementary Information S8.

Periosteal curvature is a description of the shape of the external contour of the bone surface at each slice. As such, it can give a fine‐grained description of the entheseal markings present on a bone. We use the elliptical Fourier descriptor of the surface curvature, after Morimoto et al. (2011, 2018) using the formulae described by Kuhl and Giardina (1982).

Here a closed line (*L*) has the *x* and *y* coordinates of the line point expressed as functions of a total path length *t*.

$$L=\left(x\left(t\right),y\left(t\right)\right).$$

We then decomposed the *x* and *y* coordinates separately using Fourier analysis using the following equations:

$$x\left(t\right)=A_{0}+\sum_{n=1}^{N}a_{n}\cos\mathrm{nt}+\sum_{n=1}^{N}b_{n}\sin\mathrm{nt},$$

$$y\left(t\right)=C_{0}+\sum_{n=1}^{N}c_{n}\cos\mathrm{nt}+\sum_{n=1}^{N}d_{n}\sin\mathrm{nt}.$$

Once these are obtained, a coefficient of surface curvature, *k*, is calculated:

$$k\left(t\right)=\frac{x^{\prime }\left(t\right)y^{\prime \prime }\left(t\right)-y^{\prime }\left(t\right)x^{\prime \prime }\left(t\right)}{{\left[{\left\{x^{\prime }\left(t\right)\right\}}^{2}+{\left\{y^{\prime }\left(t\right)\right\}}^{2}\right]}^{3/2}}$$

These coefficients are then assembled into a matrix. Areas of high curvature (e.g., spikes) will have high values of *k* (either positive or negative, depending on whether curvature is concave or convex). Areas of low curvature (i.e., tending toward a flat line) will have values of *k* closer to zero. The code describing this is available as Supplementary Information S9.

#### 1.3.3 Size standardization of morphometric maps and minimization of intermap distance

As the resulting projection is a 2D matrix, the underlying data can be subjected to standardization and statistical analysis. There are several options for this, of which we shall describe the three most popular one. All rely on an equal number of samples between all objects of interest, that is, the same number of slices are sampled and in each slice, the same number of measurements are taken.

##### Option 1

Morimoto et al. (2011) suggested standardization of the matrix to its median value. The matrix elements then undergo a discrete Fourier transform using the below formula:

$$\left(k\right)=\sum_{j=1}^{n}X\left(j\right)W_{n}^{\left(j-1)(k-1\right)},$$

where,

Wn=e−2πin

is one of *n* roots of unity (both equations from Frigo & Johnson, 1998).

This transform returns both the complex and real component of each vector. The complex component of the vector is discarded for subsequent calculations. The Fourier‐transformed matrices are then compared to their group means (e.g., for age group 0–3 months, the mean of all matrices) and the matrices undergo a circular shift (i.e., columns are moved from the start of the matrix to the end) in order to minimize the distance from the mean. This has the effect of minimizing user error in original orientation of the stacks (Morimoto et al., 2011). One can also do this iteratively, that is, create a group mean, shift the matrices toward the mean, create a new mean of the shifted matrices, and repeat the shifting.

##### Option 2

Puymerail et al. (2013) suggested standardizing the matrix values to positive integers between 0 and 1. A thin plate spline regression following Wood (2006) is used to align all the maps.

##### Option 3

One could keep the raw measurements and not standardize the data at all. This is followed by Lacoste Jeanson et al. (2018) as they argue that these measurements are highly correlated with body mass, and to standardize in this fashion may mask intergroup differences.

### 1.4 Aims and objectives

Here we will seek to apply the technique of morphometric mapping to an ontogenetic sample of *H. sapiens* to assess both endostructural variability (through the proxy of cortical thickness) and localized periosteal curvature from 20 to 80% increments of diaphyseal length.

We seek to answer the following questions:

1. How does cross‐sectional geometry vary along the shaft during ontogeny? Is it uniform in distribution and timing or is this a highly variable process?
2. What does the local variation in cortical thickness look like when projected to a “map” and how does this vary through ontogeny?
3. How sensitive is curvature mapping in detecting inter‐group differences when applied to the periosteal surface?
4. Do our GMM analyses of humeral diaphyseal curvature indicate that distal anteroposterior curvature becomes more prevalent through adolescence, confirming the findings of Hambücken (2012)?
5. Is dense pseudo‐landmarking better at detecting differences between age groups than landmark free methods?

## 2 MATERIALS AND METHODS

### 2.1 Sample

An ontogenetic sample of humeri from the medieval urban site of Newcastle Blackgate was selected for micro‐CT scanning. This site, located in northern England, is a medieval burial ground from the later Anglo‐Saxon through to early Norman periods (Mahoney‐Swales, 2012; Niinimäki et al., 2013) with the skeletal remains of 638 individuals of which 231 are immature. For this study, intact diaphyses of humeri of 59 immature individuals were obtained representing all ages from fetal to ~18 years of age. The ages at death of these individuals were previously estimated using dental development patterns, epiphyseal fusion and/or regressions based on long bone lengths (Mahoney‐Swales, 2012). This site previously had a sample of adult humeri analyzed using pQCT (Niinimäki et al., 2013). The composition of the sample is shown in Table 1. The sexes of the individuals are unknown, as it is impossible to ascertain the sex of immature skeletal remains solely through morphological methods and a DNA work has not yet been attempted on this sample. We can, however, assume that it is a mixed‐sex sample as it derives from a community cemetery. Left humeri were preferentially selected as these were generally better preserved in this sample and to avoid having to correct for bilateral asymmetry within individuals. A number of right humeri were also scanned, however, when intact left humeri were unavailable these scans were mirrored to augment the sample size.

**TABLE 1:** Sample composition

Age group name	Age ranges	No. of individuals
Fetal/neonate	Late fetal to 1‐month post‐natal	9
Infant	1 month to 2 years	11
Young child	2–6 years	25
Older child	7−12 years	10
Adolescent	13–18 years	6

The sample composition of the study is shown in Table 1.

### 2.2 Data acquisition

All humeri were batch scanned using a Nikon‐Metris custom bay microCT scanner at the Henry Moseley X‐Ray Imaging Facility, University of Manchester. All humeri were mounted vertically in inert foam and the foam was fixed to the scanner turntable, and highest achievable resolution for each batch was used. The method of batch scanning was chosen to maximize sample throughout, because the research questions for this study did not require a voxel resolution lower than 40 μm. Samples were scanned at 50 KeV/235 μA with isotropic voxel sizes ranging from 0.0384 to 0.108 mm, on continuous scanning mode with 2001 projections per volume. Projections were reconstructed into stacks using CTPro (Nikon Ltd.) on a dedicated workstation. Due to scanning volume constraints, some of the larger bones had to be scanned in two passes, and these stacks were automatically aligned using Avizo® 9.0 (FEI/Thermo Fisher Scientific, Inc.). Individual bone stacks were extracted using the ROI crop function in Avizo 9.0. Individual scan parameters for each specimen are available in the Supplementary Information S4.

### 2.3 Stack segmentation

Stacks were aligned vertically to their principal axes using a combination of the “moments of inertia” tool in BoneJ 1.4.2 (Doube et al., 2010) and “Reorient3_TP” (available from http://www.med.harvard.edu/JPNM/ij/plugins/AlignStacks.html). The stacks were oriented with the bone vertical with the coronal plane, parallel to the *y*‐axis. Each complete stack was then resampled to 200 equal slices the transverse plane for the whole diaphyseal length, giving 0.5% increments using the resample tool with spline interpolation in Avizo 9.0. (For our analyses we were only interested in the 20–80% margin but this was the simplest method of obtaining this subsample.) This resulted in very few artifacts from partial volume averaging (a known problem with downsampling of CT data; Abel et al., 2012) (Supplementary Figure S1). In older specimens where the epiphyses with the articular processes were fused to the diaphysis, scans were cropped at the epiphyseal margins, to ensure comparability over all age groups.

Images were semi‐manually segmented using a Wacom Bamboo® (Wacom Co.) graphics tablet and the segmentation editor “Edit label field” in Avizo® 9.0. Slices were thresholded using the magic wand tool and inner contours were manually corrected. This approach was taken as fully automated routines such as in Zebaze et al. (2013) or Buie et al. (2007) it did not yield satisfactory results. This is probably due to the fact that these algorithms were all developed using adult bone, whereas in immature bones the boundaries between trabecular and cortical bone are often diffused and para‐cortical bone is also present, especially in younger age groups. All holes caused by nutrient foramina or postdepositional cracks in the bone were manually filled in, using the surrounding internal and external contour as a guide.

### 2.4 Processing of segmented stacks

Using a series of image processing routines written in Matlab, we were able to extract the following data from whole segmented Micro‐CT stacks: Cortical thickness at regular radial intervals per slice, periosteal surface curvature, biomechanical properties, and pseudo‐landmarks.

Firstly, conventional biomechanical indices were analyzed to establish if differences are discernable between groups. These indices were automatically quantified at every 0.5% longitudinal increment of the diaphysis and a secondary aim was to see if the increment locations usually quantified (e.g., 20%) were actually of utility, or if the analysis of different locations along the bone shaft would be more useful.

Secondly, mapping of the thickness of cortical bone was undertaken, again to track change throughout development and to see if differences between age groups were statistically significant. Significant results could have a bearing on helping to narrow down age ranges for fragmentary juvenile material.

Thirdly, analysis of curvature of the external periosteal contour was undertaken to track the development regions of high curvature to see if the contours reflect muscle attachment sites and to see if any significant correlations exist between this and cortical thickness.

Finally, a GMM analysis of the entire diaphysis using coordinate data of the periosteal contour was undertaken, to compare the utility of this versus our more landmark free approach. It also gives an alternative, GMM analysis of the proximal–distal curvature of the humerus.

After segmentation, each stack of images was analyzed using a custom routine written in Matlab (Mathworks Ltd) by WIS and TO'M which characterized the thickness of the cortex in the segmented stacks. Our method for characterizing thickness broadly follows that of Bondioli et al. (2010), as the long bones of interest in this article are broadly cylindrical in cross‐section and can be measured using one radial line from the centroid, rather than a point orthogonal to the tangent at a point on the external surface as in Morimoto et al. (2011). We, however, used the centroid position for each slice, rather than the medial line of the whole bone. This was also done to reduce computational complexity, although we do acknowledge that a tangentially based thickness measure may be more appropriate for bones such as ribs, which are much more ellipsoid in cross‐section.

The Matlab code was set to only sample slices between the 20 and 80% margin (i.e., 120 slices).^1^ Each slice had 360 measurements taken in a clockwise direction from an automatically defined centroid and the following outputs were produced: Matrix of raw thickness values; slice based inner coordinates; slice based outer coordinates; XYZ array of inner coordinates; XYZ array of outer coordinates; subsamples of XYZ coordinates (subsampling was user defined); heat map of thickness matrix, both labeled and unlabeled. The routine also automatically extracted the following biomechanical parameters for each slice: total area; percentage cortical area; *I*_max_/*I*_min_; *I*_*x*_/*I*_*y*_; and *J*. The Matlab code for this and thickness calculations are available in Supplementary Information S8.

To define external curvature, another routine was written in Matlab using the broad definitions provided in Morimoto et al. (2011). Here, a folder containing the CSV files of outer points was chosen interactively. The routine then applied an elliptical Fourier smoothing on the coordinates to remove noise in the data, and the curvature coefficient, *k* (after Kuhl & Giardina, 1982) was defined per point. Outputs from this routine were: Heatmap of *k* matrix; CSV file of *k* matrix; 2D graph of original contour points; 2D graph of smoothed points (the latter help with spotting accidental oversights in alignment or segmentation). The Matlab code for this is available in Supplementary Information S9.

**FIGURE 1:**
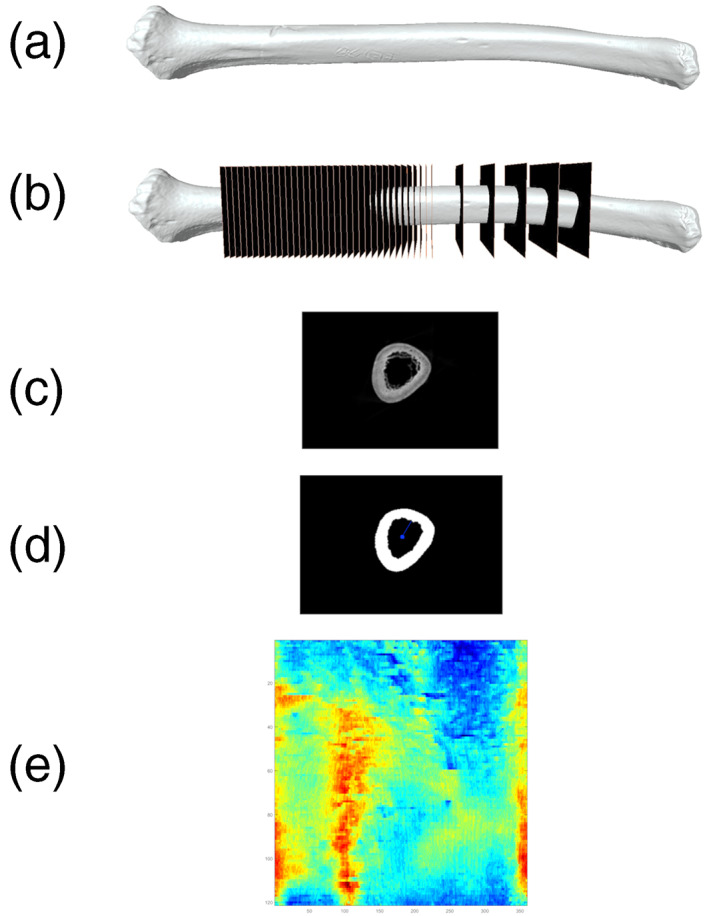
Workflow. (a) Scan bone, (b) reorient and resample stack, (c) segment resampled slices, (d) measure segmented slices, and (e) generate heatmap

An easy to digest summary of the above workflow is shown in Figure 1.

### 2.5 Analysis of data generated

For conventional biomechanical indices, group means and standard deviations were calculated. Coefficients of variation for each 0.5% increment in length were also calculated to establish positions at which differences between groups could be discerned. For percentage cortical area, a measure of the “spike” in the data, full width at the half maximum height (FWHMH) of the data was also measured (Weisstein, 2018).

For thickness based morphometric maps, all thicknesses were standardized in size before further analysis. In this case, we have decided to size standardize using the method outlined by Puymerail et al. (2013) and did not shift the matrices. This resulted in a process which is easy to both understand and to replicate. The decision made not to shift the matrices, either through the circular shift technique or through a spline, was because the stacks had been oriented prior to segmentation and the subsequent shifting of matrices removed this homology. This is probably due to the process of cortical drift in young specimens where remodeling occurred at different speeds. The code for our size standardization is in Supplementary Information S10. We also repeated the caution of Morimoto et al. (2011) that this technique does not presume perfect point‐to‐point homology among specimens; it is used to analyze variation of global patterns around and along entire diaphysis. For curvature maps, a size standardization step was not necessary as the data was already at the same scale.

The matrices were then combined by reshaping to one line each and adding to an overall matrix. This routine was also written in Matlab and is available as part of the supplementary material (Supplementary Information S11). Principal components analysis (PCA) of the matrices was conducted in Matlab. Subsequently, the principal component (PC) scores were subjected to linear discriminant analysis (LDA) in PAST version 3.25 (Hammer et al., 2001).

Although Puymerail (2013) and Morimoto et al. (2011) both suggested differing decomposition methods to ease subsequent data analysis, as the data have already been standardized, it is no longer technically necessary to do this, and separation of groups was actually improved by removing this step.

To establish how different the analysis of periosteal curvature based on Fourier decomposition would be to that of a more familiar GMM analysis, the same external coordinates were subjected to Procrustes superposition in the R package “Morpho” (Schlager, 2013). Discriminant function analysis (DFA) of the resulting eigenvectors was analyzed in PAST V3 (Hammer et al., 2001). Extremes of the plotted PC axes were also generated using the package Geomorph (Adams & Otarola‐Castillo, 2013).

## 3 RESULTS

### 3.1 Conventional biomechanical parameters

#### 3.1.1 Percentage cortical area

**FIGURE 2:**
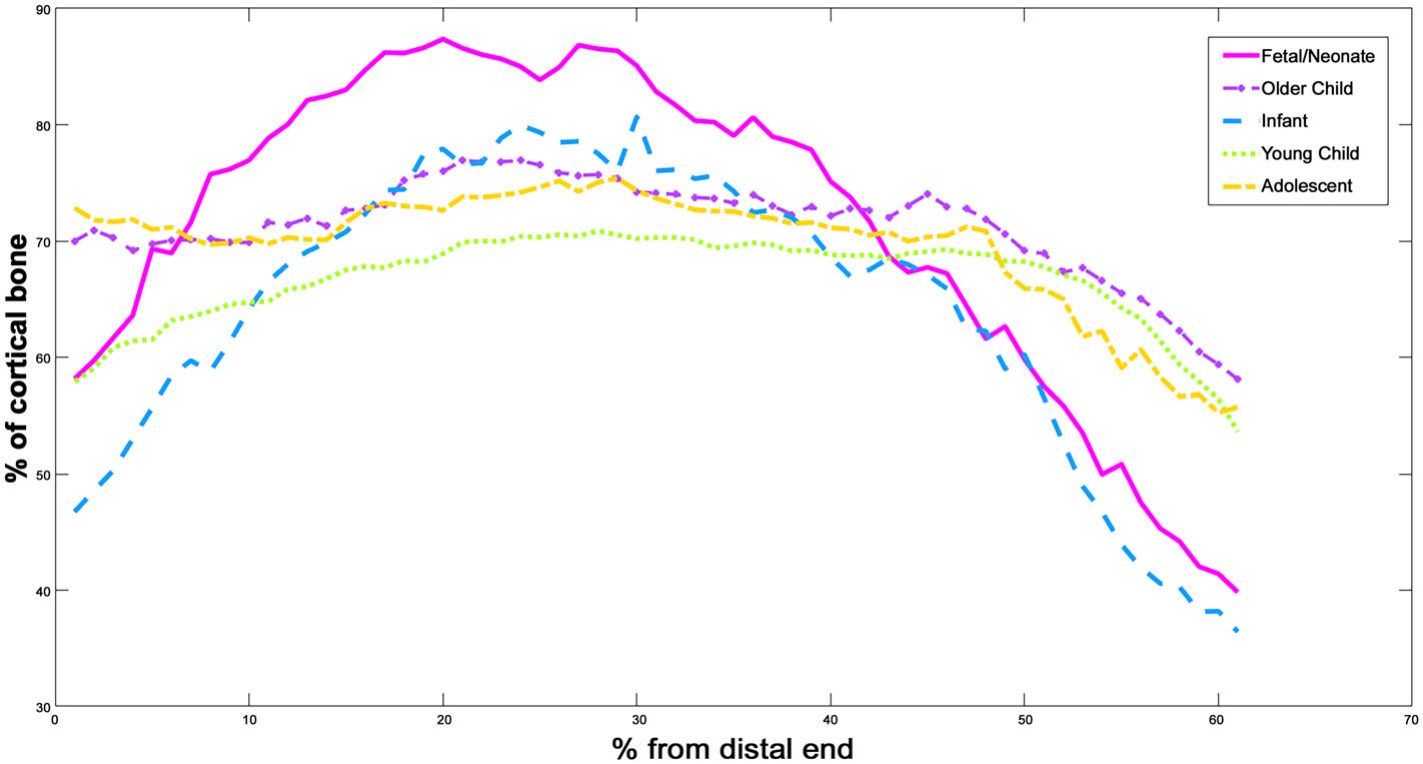
Cortical area of humeri—all age groups

Percentage of cortical area provides a standardized measure of areal bone density. Here (Figure 2), it can be seen that a peak of cortical bone volume is achieved between 40 and 60% increments in all age groups but that this peak more marked in the fetal/neonatal and early walking individuals, as evidenced by Figure 2 and Table 2, which shows the FWHMH of the data. Cortical bone volume is highest in the neonatal age category, then falls successively during infancy before rising progressively during childhood and adolescence.

**TABLE 2:** Full width half‐maximum height (FWHMH) of % cortical bone

Age group	FWHMH
Fetal/neonate	51
Infant	44
Young child	53
Older child	51
Adolescent	43

#### 3.1.2 *I_x_*/*I*_*y*_, the circularity of cross‐section

**FIGURE 3:**
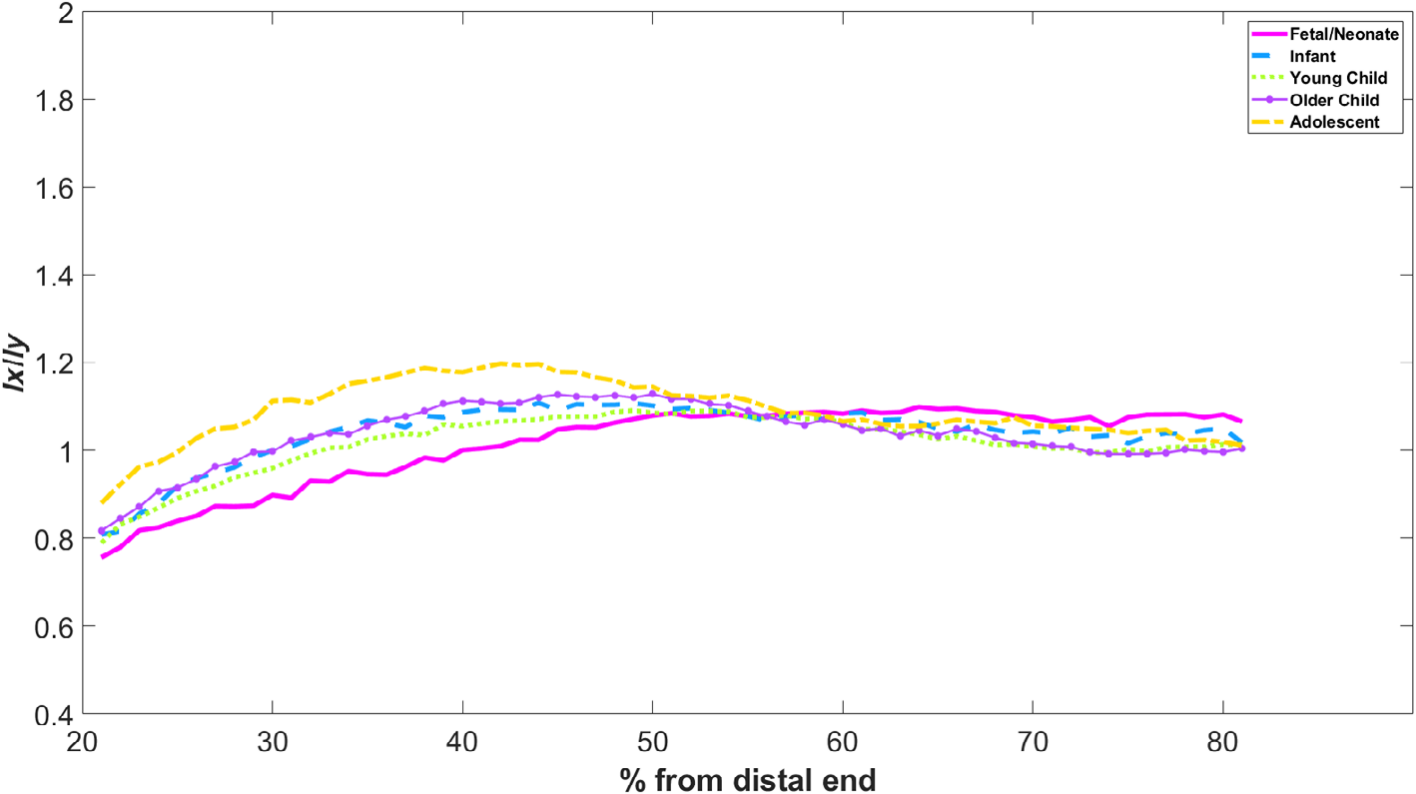
*I*_*x*_/*I*_*y*_ of humeri—all age groups

This measurement is of how close the cross section is to that of a perfect circle (where *I*_*x*_/*I*_*y*_ would be one) or whether it is more ellipsoid. Values below 1 are where the ellipse is more expanded in the anteroposterior direction, and values above one show expansion in the mediolateral direction. Figure 3 shows this over the 20–80% margin for mean values of each group. Smoothing was achieved by a locally selecting 20% of points to influence each point's smoothing (Cleveland, 1979).

Deviation from circularity varies considerably along the diaphysis. In the earliest stages, the shaft is much more ellipsoid in the mediolateral direction at the 20% margin and decreases constantly, resulting in a relatively circular midshaft. A more antero‐posteriorly ellipsoid shape is observable in the proximal humerus. This is most marked in the fetal/neonatal stages, but the infant crawling group also exhibits this shape characteristic in the proximal portion. The infant walking, young child, and older child stages broadly follow the pattern but have a fall‐off in ellipsoidal form toward the proximal margin. This repeats itself in the adolescent stage, but at the 40% margin, the bone reaches a peak of non‐circularity in the anteroposterior direction.

#### 3.1.3 *I*_max_/*I*_min_, evenness of rigidity

**FIGURE 4:**
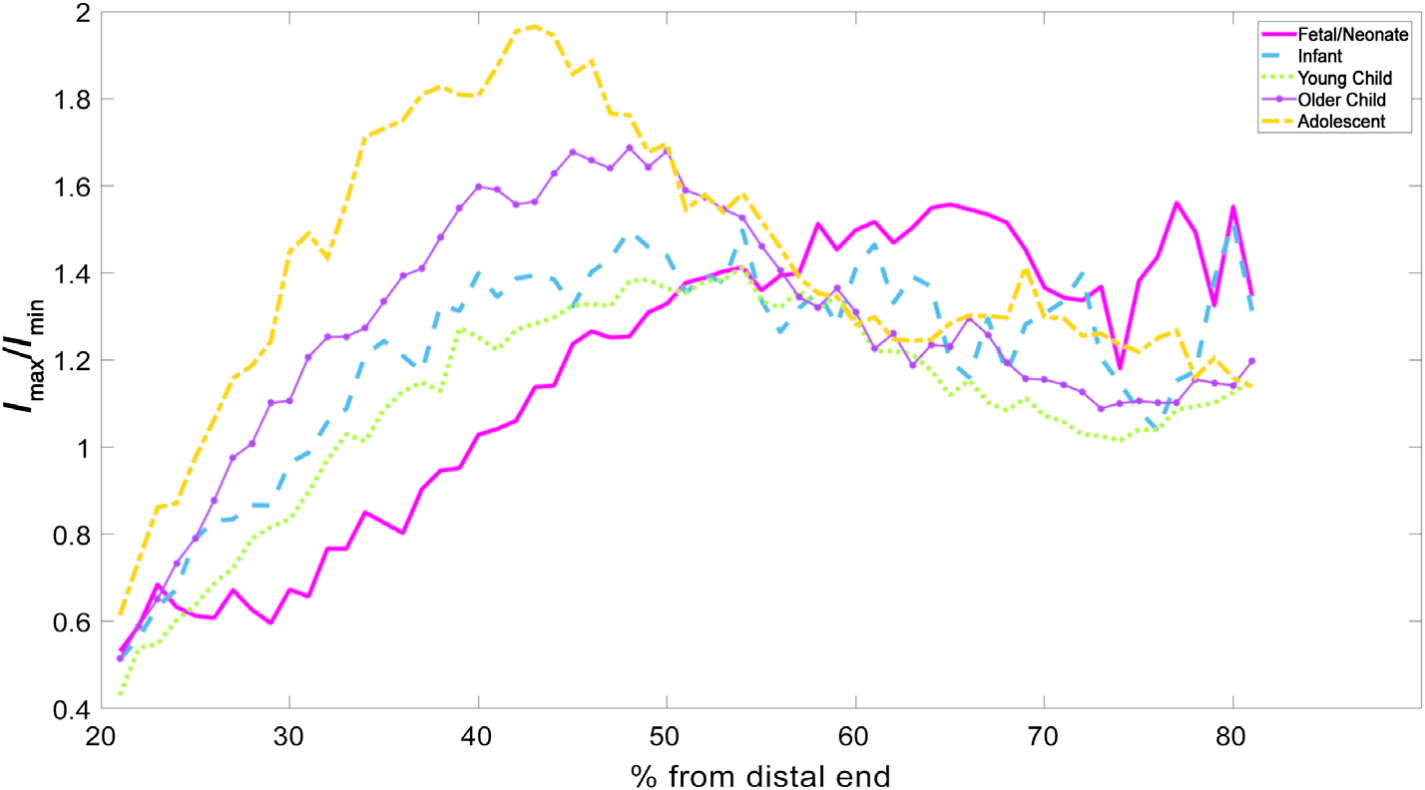
*I*_max_/*I*_min_ of humeri—all age groups

The patterns observed here are very similar to *I*_*x*_/*I*_*y*_ (Figure 3) but more exaggerated for *I*_max_/*I*_min_. As such, the discussion of *I*_*x*_/*I*_*y*_ (Figure 4) holds for this as well. To ease comparison, both sets of variables (Figures 3 and 4) are displayed with the same scale *y*‐axis.

It is difficult to compare the distribution of the *I*_max_/*I*_min_ data statistically between the groups as they all show different modes of distribution. For two groups (fetal/neonate and infant crawling), the shape of the graph makes measuring the FWHMH impossible. In lieu of this, group mean maximum heights, with the margin at which they occur, are provided in (Table 3).

**TABLE 3:** Maximum values for *I*_max_/*I*_min_ and the margins at which they occur

Age group	% From distal	Mean	*SD*
Fetal/neonate	64	1.56	0.68
Infant	53	1.52	0.60
Young child	53	1.40	0.38
Older child	44	1.70	0.66
Adolescent	42	1.97	0.52

#### 3.1.4 *J*, torsional rigidity

**FIGURE 5:**
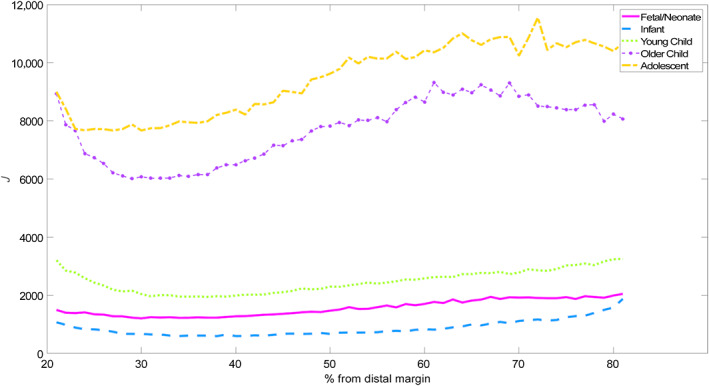
*J* of humeri. The key is the same as for Figures 2, 3, 4

In all age groups, *J* is at its lowest between the 30 and 40% margin (Figure 5). All groups are very slightly bimodal with a peak at the end closest to the distal margin, and broadly increase the second moment of the area toward a second peak, at or near the proximal margin. For visual ease, the results for older child and adolescent groups are presented on a separate graph of a different scale (Table 4).

**TABLE 4:** *J* mean values per group

Age group	% Margin‐minimum	*J* mean	*SD*
Fetal/neonate	29	1,210.3	2,881.8
Infant	37	597.7	420.8
Younger child	35	1946.3	1,031.3
Older child	28	6,010	3,573.6
Adolescent	29	7,668.5	4,704.5

**FIGURE 6:**
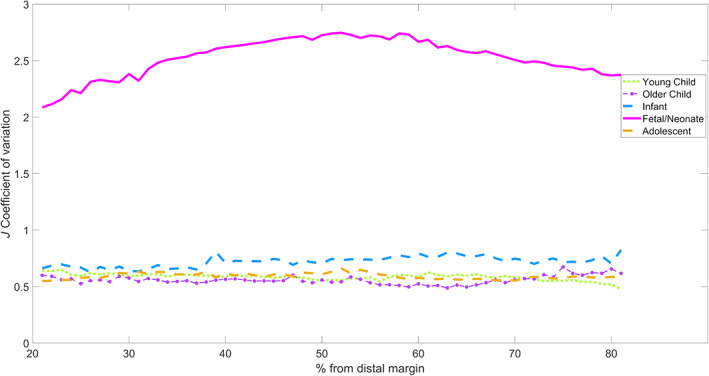
Coefficient of variation: *J*

If we look at the coefficient of variation within groups (Figure 6), the following patterns become evident. Most groups increase in variation toward the midshaft and drop off in variation toward the proximal and distal margins. The exceptions are adolescent, which decreases over the entire range and older child, which remains fairly flat. As can be seen from the graph, the data have a lot of noise in it, which makes locally weighted smoothing (LOESS) fitting only effective for a minority of age groups. This suggests that the midshaft is a more effective point to take measurements, although only for younger age groups.

### 3.2 Cortical thickness

**FIGURE 7:**
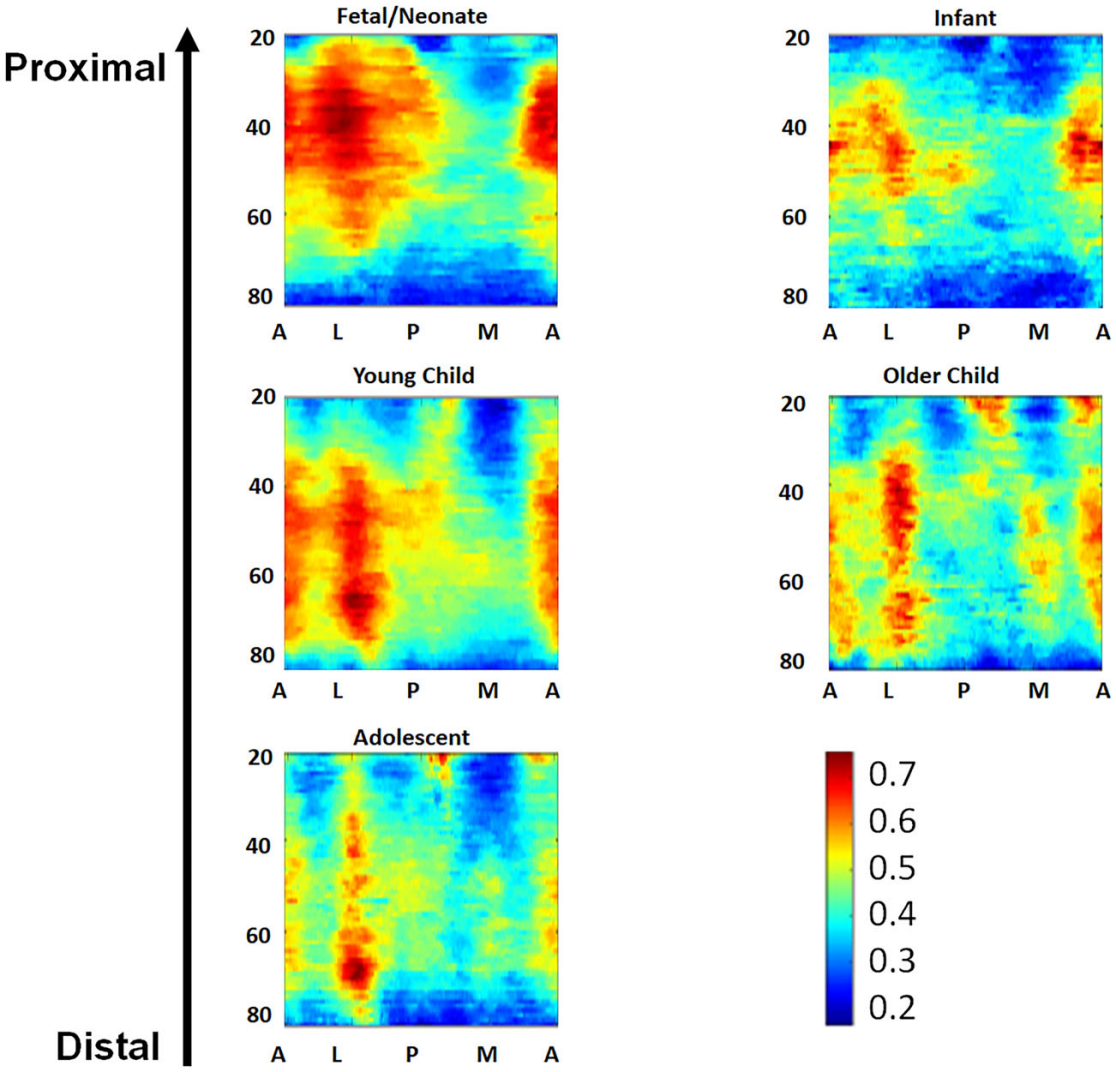
Mean surface curvature maps. Red–blue scale of increasing thickness of cortical bone. All show the 20–80% range, with 20% at the top

The standardized mean heat map for each group is presented in Figure 7. Here, a broad trend toward the organization of the internal bone structure can be seen, with the neonates appearing to have a unimodal distribution of cortical thickness across the map, whereas the later stages appear to be more complex/multimodal. In younger age groups, especially neonatal individuals, there are two main regions of diffusely increased thickness toward the distal margin. In older age categories, there are four regions of increased thickness—one located distally and three proximally. Individual maps are available in the Supplementary Information S1. When “re‐wrapped” around the bone's diaphysis in adolescents, this patterning resembles the pattern of entheses on the bone; however, the overall correlation between cortical thickness and localized surface curvature is weak.

**FIGURE 8:**
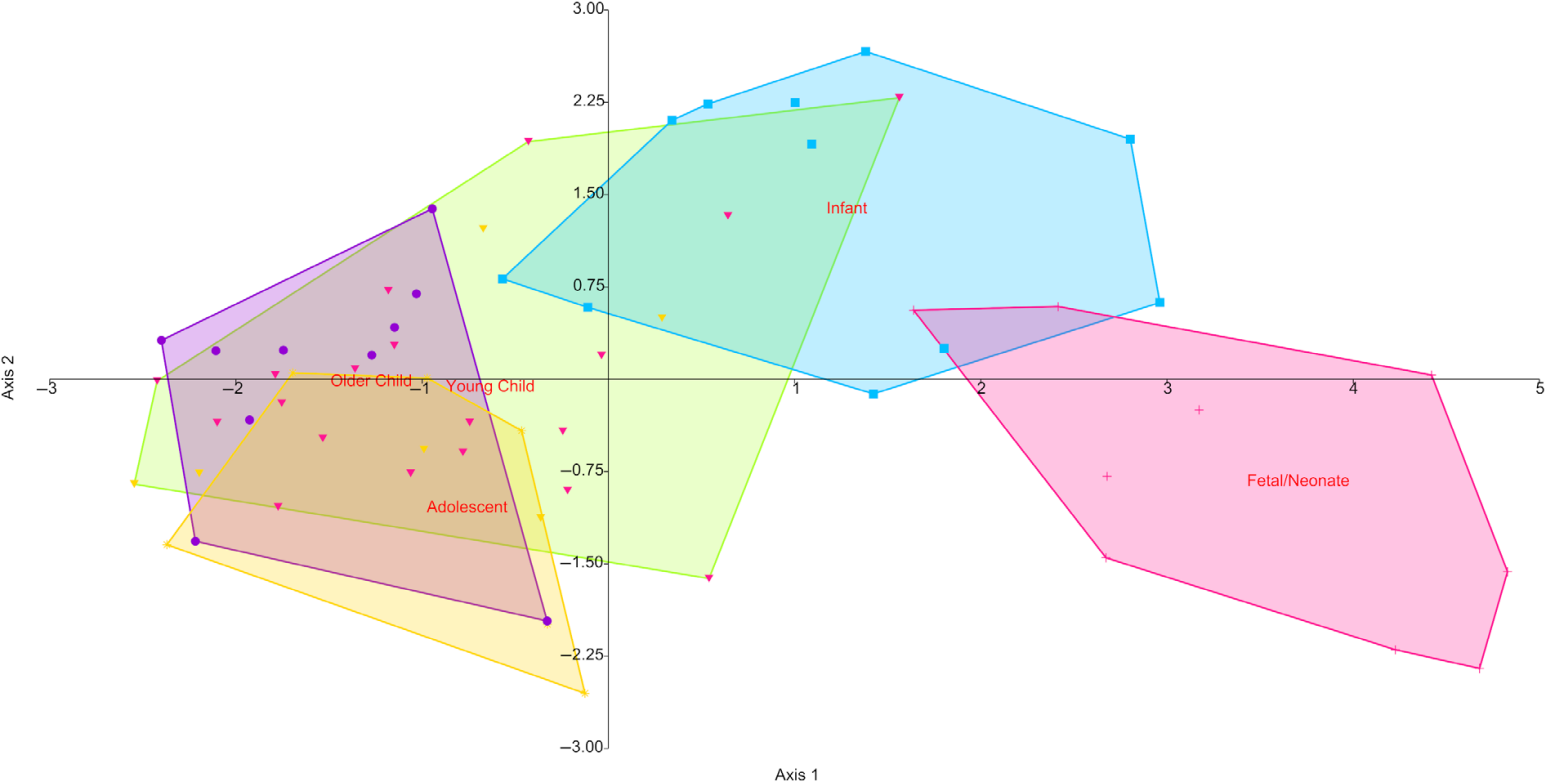
Discriminant function analysis of thickness maps, showing PC1 versus PC2. PC, principal component.

Results for DFA (Figure 8) show a good separation of groups. Axes 1–3 explain 83.33% of all variation. Fetal/neonate and young child both form visually distinct groups; however, overall the classification of individuals is poor, with only 12.07% of individuals being correctly classified on jackknifing. Results for Axes 1–3 of PCA are displayed in Supplementary Figure S1 for cortical thickness. In the PCA, fetal/neonate, infant crawling and infant walking groups all separate clearly from the rest of the groups. The first three components accounted for 85% of all variance. Graphs of PC1 versus PC2 and PC2 versus PC3 are provided as Supplementary Figure S5. Using the scores from DFA rather than from PCA helps the eye to see grouping patterns more clearly, although, with these analyses of high‐resolution data, a different grouping technique such as between group PCA (as implemented by Mitteroecker & Bookstein, 2011) may be more effective (there is, however, debate about the effectiveness of between‐group PCA; Bookstein, 2019; Cardini et al., 2019). In a situation like in this article, where there is a much larger number of variables than specimens between group PCA may result in observing patterns in data were there are none. As such, we err on the side of caution and use traditional PCA.

### 3.3 Periosteal curvature

**FIGURE 9:**
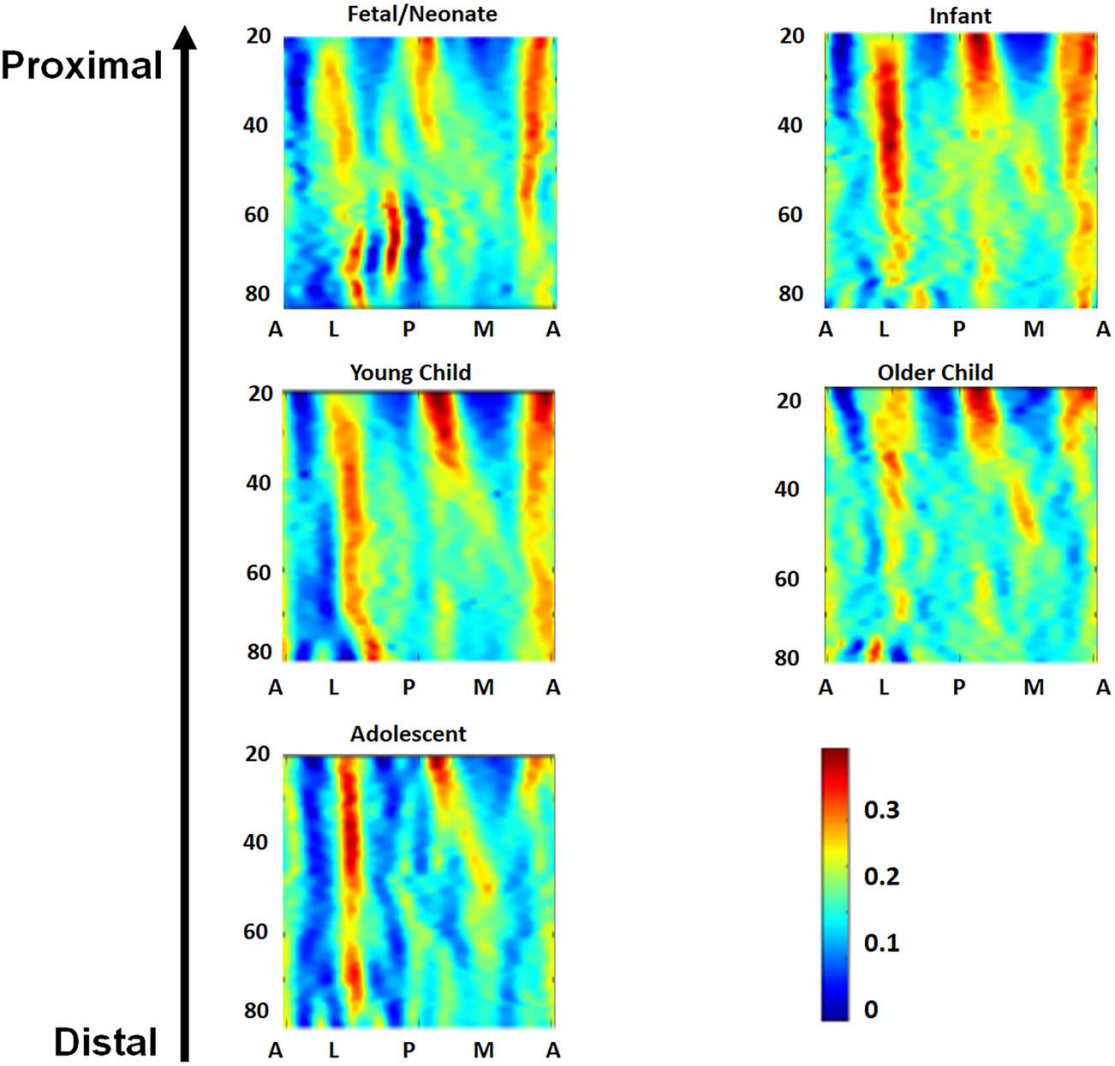
Mean surface curvature maps. Red–blue scale of increasing acuteness of surface curvature. All show the 20–80% range, with 20% at the top

**FIGURE 10:**
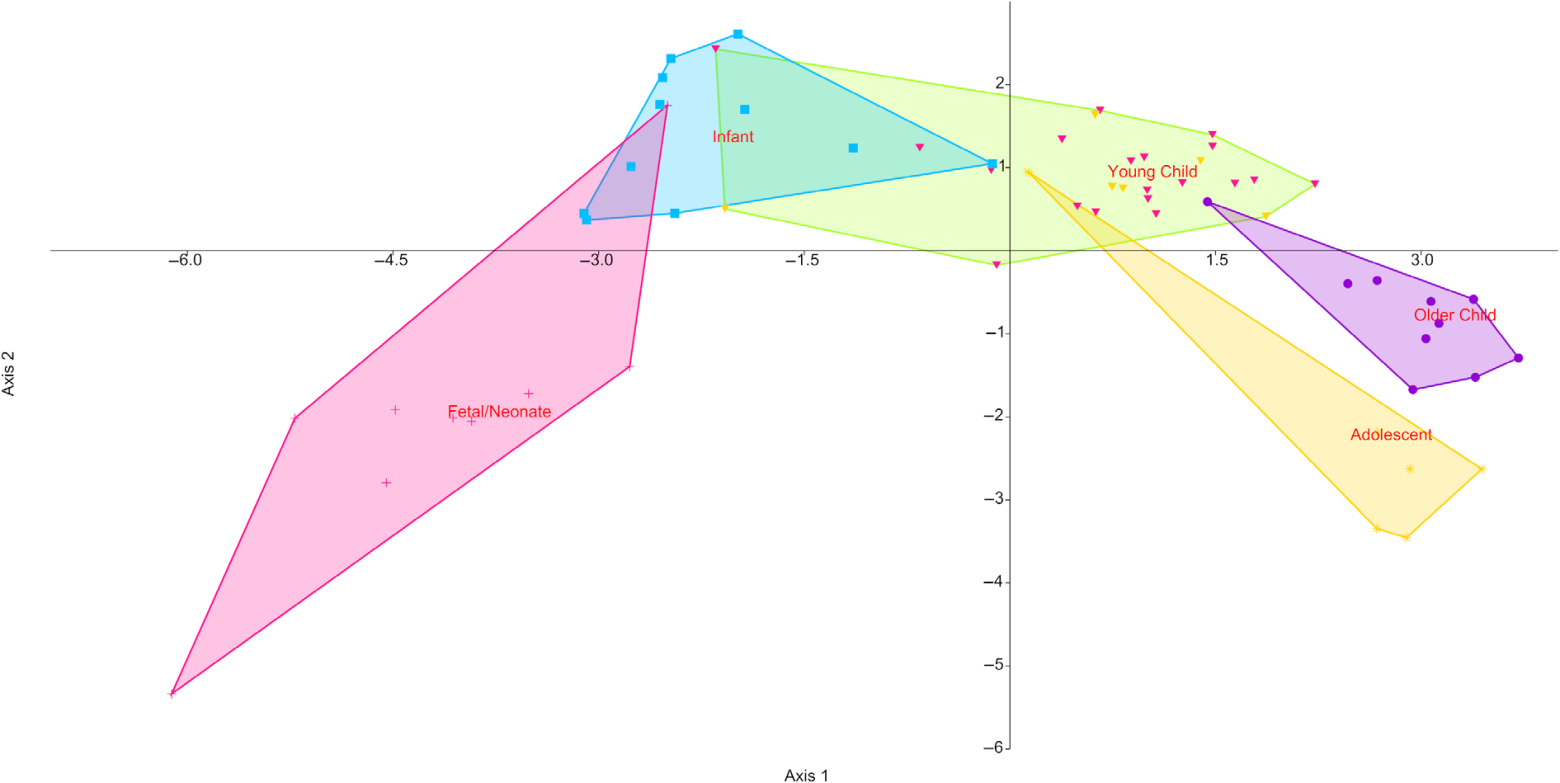
Discriminant function analysis of the surface curvature of the humerus

The surface curvature maps for mean values in each age group are shown in Figure 9. Here, three longitudinal bands of increased curvature, are visible, which are most marked toward the distal end of the bone. These regions correspond well to the overall shape of the developing humerus, which has a rounded triangular cross‐section with three broad ridges located anteriorly, posteromedially, and posterolaterally (the latter two extend to the supracondylar ridges). Individual maps are available in the Supplementary Information S2. Results for Axes 1 and 2 of LDA are displayed in Figure 10. Graphs of PC1 versus PC2 and PC2 versus PC3 are shown in Supplementary Figure S6.

Regarding thickness, “Infant walking” bones group very differently to the rest of the age classes, and infants only just border the other groups. In the case of external surface curvature, the fetal/neonatal and infant crawling groups are very different from the other age groups.

Correlation of raw scores between periosteal curvature and cortical thickness was not statistically significant at *p* = .05, using Pearson's correlation coefficient.

### 3.4 GMM analysis

**FIGURE 11:**
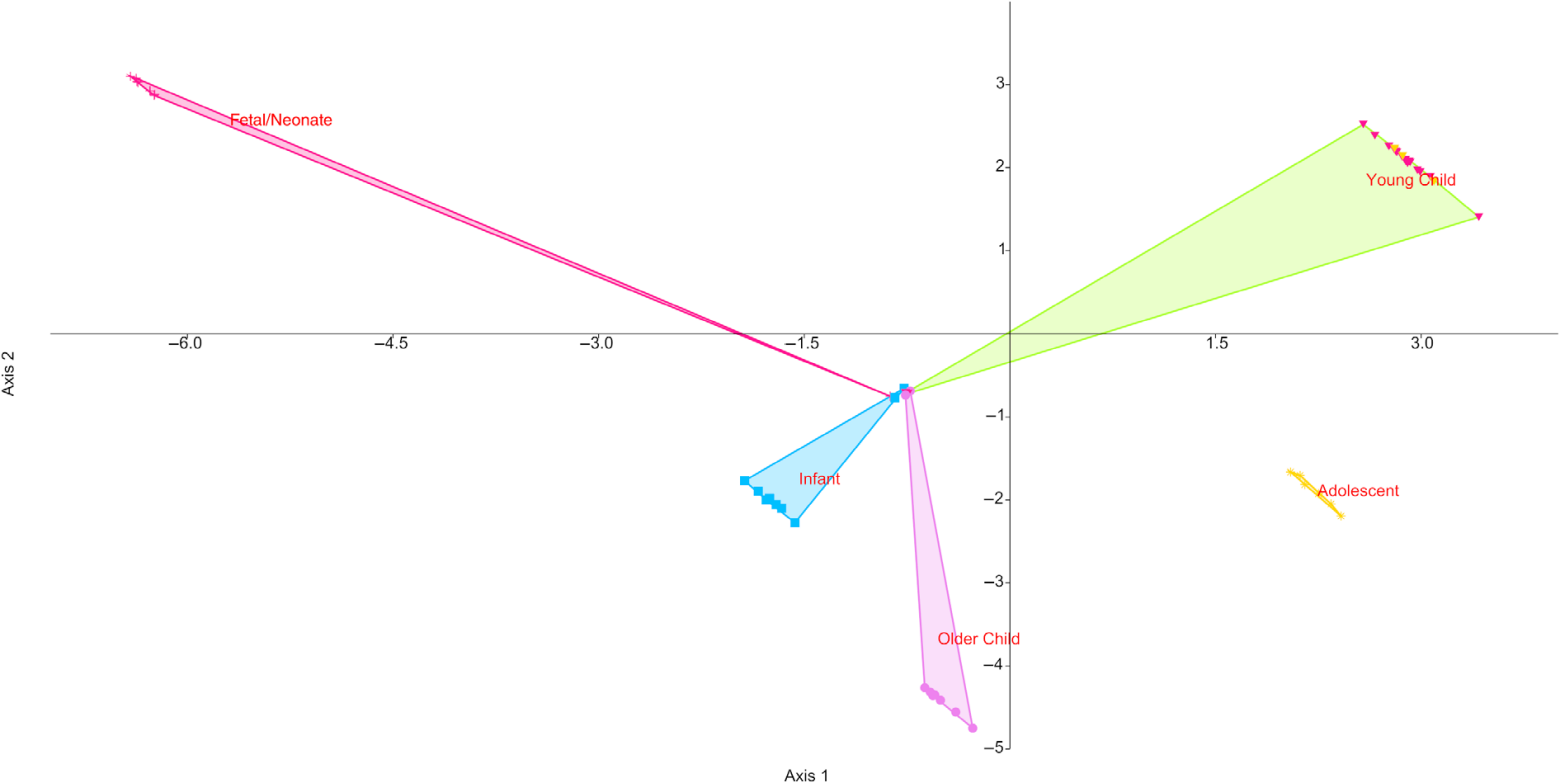
Discriminant function analysis of geometric morphometrics of humeral periosteal surfaces, showing PC1 versus PC2

**FIGURE 12:**
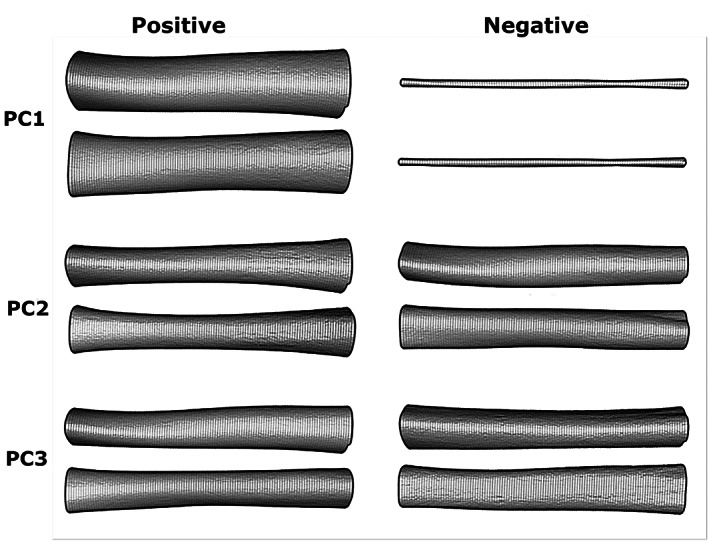
Visualizations of extremes in shape variation for the first three principal components. Note that these have all been scaled to the same length. Visualized using Geomorph version 3.07

GMM analysis of the diaphyses separated fetal/neonatal, infant, and adolescent age groups fairly confidently. A plot of discriminant analysis of the PC scores of this data is shown in Figure 11. Extreme shapes from each of the first three principal components are shown in Figure 12. What can be seen immediately is that the GMM analysis was more effective at analyzing longitudinal curvature, rather than the periosteal surface curvature analyzed using maps.

The first three principal components accounted for 96.6% of the total variation. The PCA was not very good at separating the differing groups (Supplementary Figure S7) and LDA only correctly classified 19.6% of specimens on jackknifing. This suggests that this dense sampling of semilandmarks is excessive for the determination of group affiliation.

## 4 DISCUSSION

### 4.1 Conventional biomechanical indices

For percentage cortical area, *I*_max_/*I*_min_ and *I*_*x*_/*I*_*y*_, bones tend to exhibit peaks between 40 and 50% margins, with different peaks for each age group. It must be emphasized, however, that variation, both along the bone and between groups, even in our moderate size sample, is large and not consistent.

With *J*, resistance to torsion, all age groups see peaks at the proximal and distal ends, which is evidence of buttressing against strain. As the ends of the humerus are where we see the greatest density of muscle attachments and are close to articular surfaces where stress may be concentrated, this is not a surprising finding. The 30–40% margin tends to be the area of least resistance to torsion. This would suggest therefore that analyses which only examine the 40% or 50% margin (e.g., Cowgill, 2010; Trinkaus et al., 2002) may actually miss out on areas of the bone which are both potentially of interest for inter‐group discrimination and biomechanically significant.

Of interest, is that percentage cortical area is highest in fetal/neonatal individuals, and then rapidly falls to its lowest in infants. It then progressively increases again in children and then adolescents. This suggests that the medullary cavity is very small in the prenatal period, a feature also noted by Cambra‐Moo (2014) (though they only included a single neonatal specimen in their study). This refines the findings of Cowgill (2007, 2010) and may be due to our inclusion of paracortical bone, which is easier to identify in μCT images than biplanar X‐rays as well as greater subdivision of the age groups. This improves our understanding of the early growth of human long bones. There is a rapid deposition of cortical and paracortical bone in early embryological/fetal stages (as can be seen from Carnegie stage 15; De Bakker, 2018) which provides a “scaffold” for muscles and tendons to attach to, which is more stable than cartilage. This overproduction of bone during gestation has also been observed (albeit for trabecular bone) in the vertebrae (Acquaah et al., 2015) and the femur (Milovanovic et al., 2017). It has been suggested that this overproduction is probably a product of rapid endochondral ossification. Here, a large amount of bone is laid down rapidly in a genetically programmed process which ensures rapid growth, similar to the cartilage model (Milovanovic et al., 2017). Postnatally, the need to develop trabecular bone to withstand less predictable loading means that this early cortical bone is rapidly lost. It then recovers as growth proceeds and body mass increases. The fact that cortical bone occupies over 50% of bone volume for most groups suggests that it is selected for as it is the densest type of bone and able to withstand the greatest amount of force.

The covariance ratio also increases from fetus to infant, then declines. This is probably because the medullary cavity is expanding relatively in the period of rapid postnatal growth.

Overall, these results suggest that the conventional analysis of the internal growth and shape change of the humerus demonstrates a highly variable process between individuals. As the sample analyzed here is cross‐sectional rather than longitudinal (like those studied by Tanner, 1981) this high variability could perhaps be accounted for by the uncertainty in aging of individuals. Further studies using known age and sex individuals, either from clinical or osteological data, may help to clarify these trends somewhat.

### 4.2 Thickness maps

Over development, the thickness maps tend to show an increase in the organization of the internal structure of the humerus. In fetal/neonatal and infant age groups, there is very little trabecular bone, and para‐cortical bone is also present. There is a large position of cortical bone in the proximal half of the humerus on the anterior portion. This is due to a large portion of early growth being from the proximal growth plate and the lack of trabecular bone in extremely juvenile individuals.

It would appear, based on maps for older age groups, that what is generally termed “para‐cortical bone” does not all transmute to cortical bone, and in fact some becomes trabecular bone, which is also known to become more regular in structure through development (Cambra‐Moo, 2014; Carter & Orr, 1992). The concentration of cortical bone shifts toward the mid shaft in these latter groups (from “Infant Walking” stage onwards) and areas of deposition become more discrete. This is probably linked to both the redeposition and regularization of structure of trabecular bone (Cambra‐Moo, 2014; Carter & Orr, 1992). It can be observed that the shift of cortical thickness is mainly to the anterior and medial portions of the bone, which are where the triceps and brachialis both attach.

The discriminant analysis allows, in this sample, to start to distinguish the first three stages from all others. With larger samples, and more refined age estimates (either by using known age samples or refined dental aging, either histological, or radiographic); it may be possible to distinguish better between the latter age groups as well. It seems, however, that this sample is not large enough, or the group too homogenous, for intergroup divisions to be greater in these latter stages than intra‐group ones. Due to the sample composition, only the young child group is significantly larger than any other (there are no statistically significant differences between the sample sizes for the different age groups [chi‐square *p* = .43]) but distortion of the results by an overly dominant subsample is not of concern here. Some of the samples are quite small however, and more differences might have emerged if larger sample sizes were studied. Unfortunately, due to the large overlap between many of these groups and the large error in classification of samples upon jackknifing, these results should be treated with caution. It may be that downsampling of the data here may be useful to see if the grouping observed here is easier to distinguish.

### 4.3 Curvature maps

Periosteal curvature tells a slightly different story to that of cortical thickness. The external surface of fetal/neonatal bone appears to be relatively clearly defined into ridges and peaks. This is probably because development is canalized at this stage and normal fetal development in this sample progressed in a homogenous fashion. We also know from previous work that this population was relatively well‐nourished (Mahoney‐Swales, 2012) and it was a homogenous population in terms of origin, so weight can be added to this argument in this fashion. Muscle contractions in utero will have therefore made a proportionally large impact on the very delicate periosteal surfaces at this stage, but this is only an observation we have been able to make due to the adequate resolution of the scan data.

For infant and young child stages, the pattern of marking is more diffuse. One potential effect may have been the use of swaddling of infants, thought to have been common in the medieval period, rendering them fairly immobile for extended periods during early postnatal development. This is, however, speculative as we have no direct textual evidence of this being practiced in Newcastle during this time period. Another is that a large amount of individual variation in achieving standard developmental goals that are observed in all communities as infants became more mobile and independent. In older child and adolescent stages, markings become more defined, which probably indicates the assumption of regular patterns of activity for these individuals. As our sample is medieval, one must also bear in mind that individuals over the age of approximately 13 would have been assumed to be capable of, and would have been expected to participate in the full range of adult physical activities. The maps also show shallow or negative curvature in the regions where the triceps and brachialis muscles attach, suggesting that these muscles, (which act to flex and extend the arm at the elbow joint) play a dominant role in development of humeral shape.

Discriminant analysis distinguishes fetal and early crawling age groups from all the others, showing that the differences between groups are real. Again, however, the classification under jackknifing was poor and these results should be treated with caution.

### 4.4 Geometric morphometric analysis

Dense sampling undertaken in an automated and indiscriminate fashion, as here, lends itself more to a broad separation of groups and overall object shape. This suggests that this application of GMM is more effective at analyzing longitudinal curvature, rather than periosteal surface curvature.

Our results differ subtly from Hambücken's (2012) results, as we find that longitudinal curvature of the diaphysis is probably proportional to bone length, where a shorter humerus is likely to be more curved. This parallels the results found in the femur, radius and ulna in adults by De Groote (2011a, 2011b). Developmental differences in the influence of the deltoid may also have an influence, but PC1–PC3 indicate that size of the bone is a major contributing factor to overall curvature.

It can be observed, however, that fetal and neonatal bones are very separated from other groups. This is due to the relative stoutness of fetal and neonatal bones relative to length, which is distinctive to all other age groups. Indeed, infant humeri are also largely separate from other age groups as they are also stout, but in a different fashion. This is also probably due to the rapid lay down of bone observed internally during the fetal period in both this bone, the vertebrae (Acquaah et al., 2015) and the femur (Milovanovic et al., 2017).

Another question that can be asked is how homologous are the landmarks from this type of analysis? If they are not homologous, then this may violate one of the principles of GMM, which is after all, the analysis of changes in homologous structures. Stern et al. (2015) have found in mouse models that one can effectively track different portions of the bone throughout development, and that the relative position of major structures stays the same, that is, they scale allometrically. We concur with this argument as our study is limited to the same bone from the same species. How effective this is when multiple species are analyzed (as in Boyer et al., 2015) is one of considerable debate (e.g., Gao et al., 2018).

### 4.5 Use of PCA and LDA

One of the findings of this study has been that PCA and LDA analysis are only moderately effective at differentiating between different age groups. This is probably due to the high dimensionality of the data and the fact that there is high degree of internal correlation between points as this study sampled measurements extremely densely. There are two potential routes for amelioration of this problem.

Secondly, it may be that PCA and LDA are simply not appropriate tools at this level of resolution, and that alternatives need to be sought. There are multiple techniques available for this. An easy to implement solution may be between group PCA (as implemented by Mitteroecker & Bookstein, 2011, but see caution in Bookstein, 2019, Cardini et al., 2019). An alternative is to use different pattern recognition methods, such as adversarial neural networks (e.g., Nielsen, 2015; Radford et al., 2015) or more general machine learning approaches (as carried out by Püschel et al., 2018).

### 4.6 Future work

This study has demonstrated the utility of approaching a large proportion of the diaphysis using automated analytical techniques. We would, therefore, re‐emphasize the need for researchers to examine multiple sites throughout the diaphysis of the humerus in order to effectively track variation and to potentially more finely discriminate between groups. Where possible, a whole bone approach should be employed.

The most significant finding is the rapid decline in cortical bone postnatally, after excess production in utero, which concurs with finding from the vertebral column and the femur (Acquaah et al., 2015; Milovanovic et al., 2017). Both cortical thickness and periosteal curvature mapping can be related to muscular development, especially that of the brachialis and triceps. GMM analysis revealed that longitudinal curvature of the humerus is largely allometric, as previously found in adult femora, radii, and ulnae (De Groote, 2011a, 2011b).

Future research should look at different human groups, in order to establish whether the patterns observed here are more generally applicable to *H. sapiens* generally. Examination of comparative juvenile fossil samples (e.g., *H. neanderthalensis*), as well as ontogenetic series of hominoid primates (as examined for the femur by Morimoto et al., 2011, 2018), would also be fruitful. Further work will also focus on expanding this methodology to both other long bones and to other species, as well as known age samples in order to test this hypothesis of finer discrimination. The use of different pattern recognition methods, such as adversarial neural networks (e.g., Nielsen, 2015; Radford et al., 2015) or more general machine learning approaches (as carried out by Püschel et al., 2018) will also be explored, as these may be more effective for classification than conventional multivariate methods.

## AUTHOR CONTRIBUTIONS

**Thomas George O'Mahoney:** Conceptualization (lead); data curation (lead); formal analysis (lead); investigation (lead); methodology (equal); project administration (equal); software (equal); validation (equal); visualization (equal); writing – original draft (lead); writing – review and editing (lead). **Tristan Lowe:** Investigation (supporting); methodology (supporting); resources (equal); validation (supporting); writing – review and editing (supporting). **Andrew Timothy Chamberlain:** Conceptualization (supporting); formal analysis (supporting); funding acquisition (lead); methodology (supporting); resources (equal); supervision (equal); validation (equal); writing – review and editing (equal). **William Irvin Sellers:** Conceptualization (equal); formal analysis (supporting); methodology (supporting); software (equal); supervision (equal); writing – review and editing (equal).

## Supporting information


**Appendix S1** Supporting Information.

## Endnote

^1^
As a feature of this code, the user has the option to apply this as a global option, within the script itself, or they can activate a dialogue box that will ask for the preferred range each time.
