# Supplementary material for: Endostructural and periosteal growth of the human humerus
Source: Anat Rec (Hoboken). 2022 Aug 25;306(1):60–78. doi: 10.1002/ar.25048 (PMC10086792; doi:10.1002/ar.25048)
Supplement: Supplementary file 1 — Appendix S1 Supporting Information. [file AR-306-60-s001.docx]

### S1. Example of downsampling


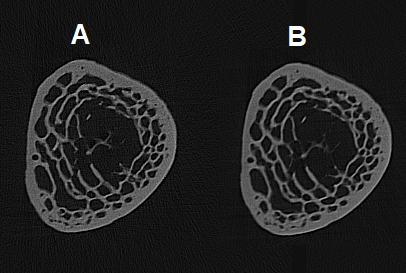


Effects of downsampling. A: Original resolution slice, B: Downsampled slice. As downsampling was in the Z plane, the downsampling had little effect on X-Y resolution.

### S2. Cortical thickness maps for all specimens


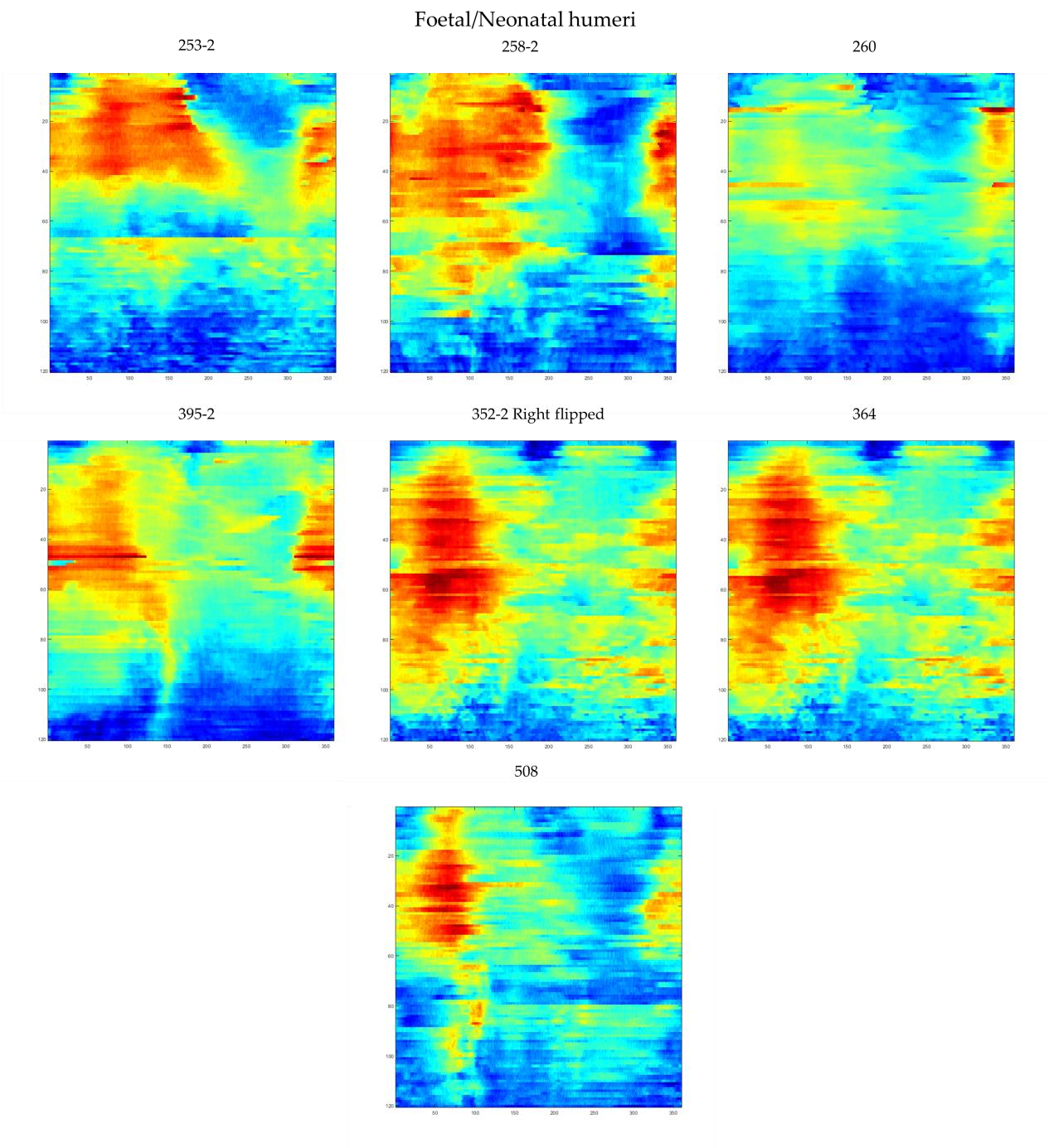


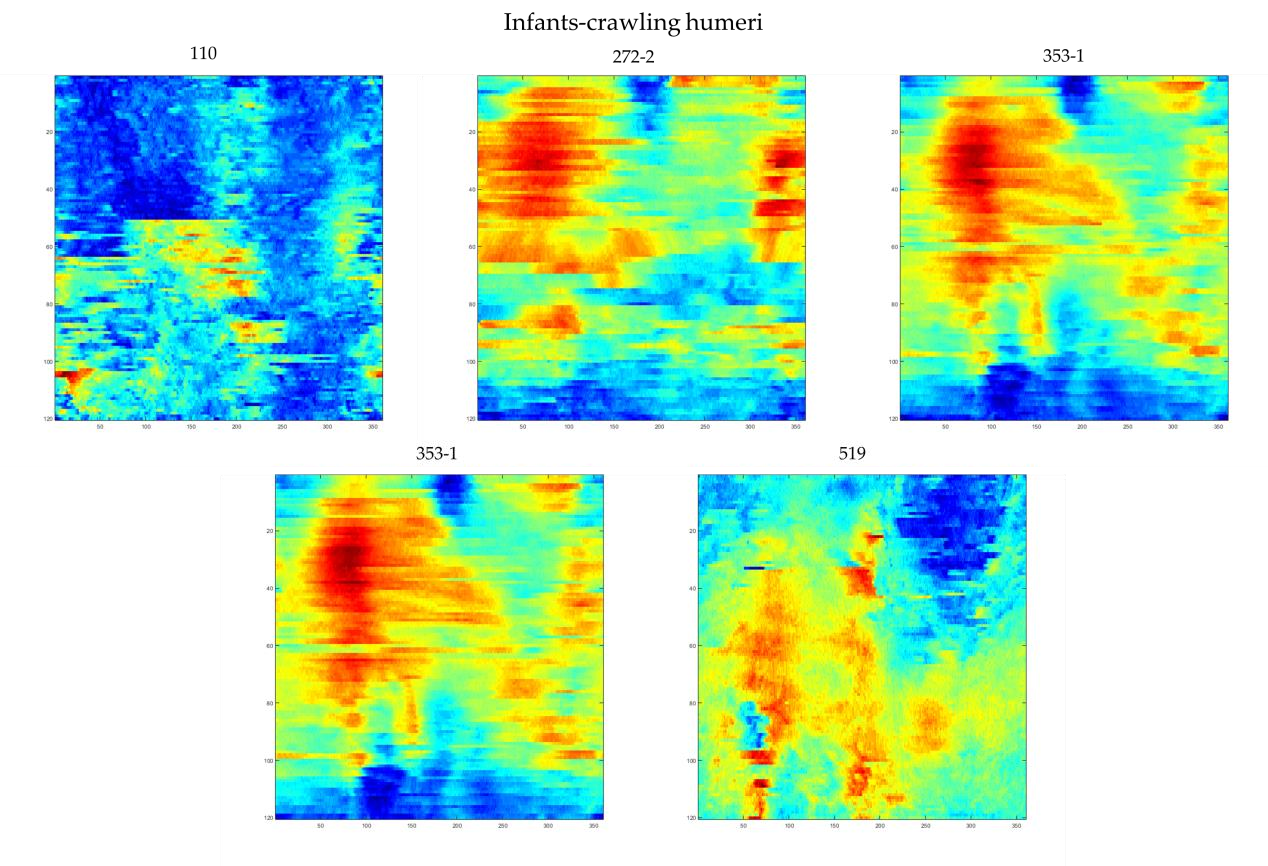


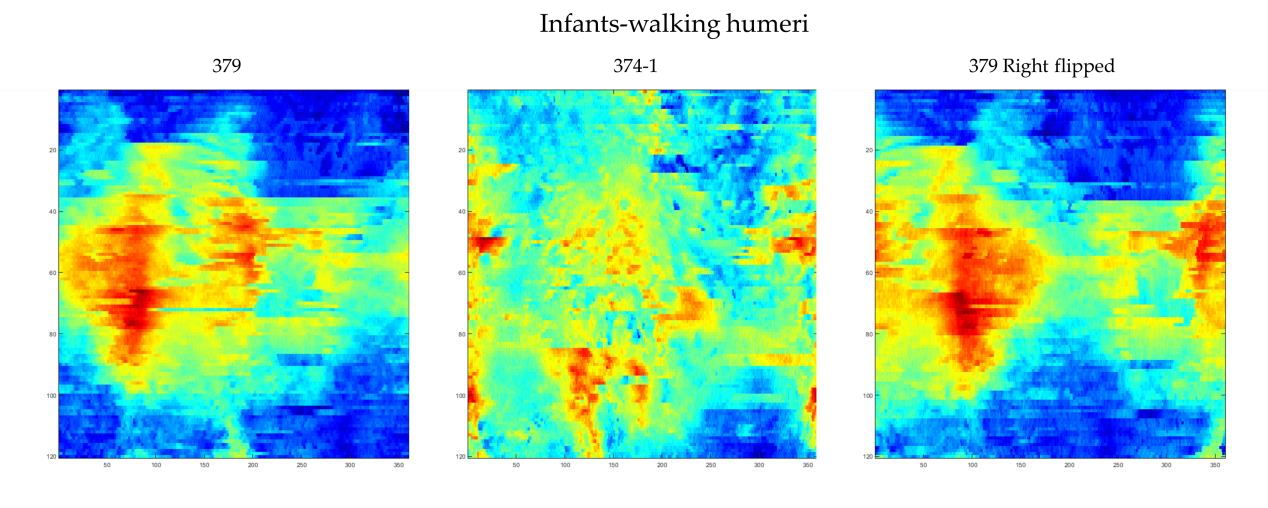


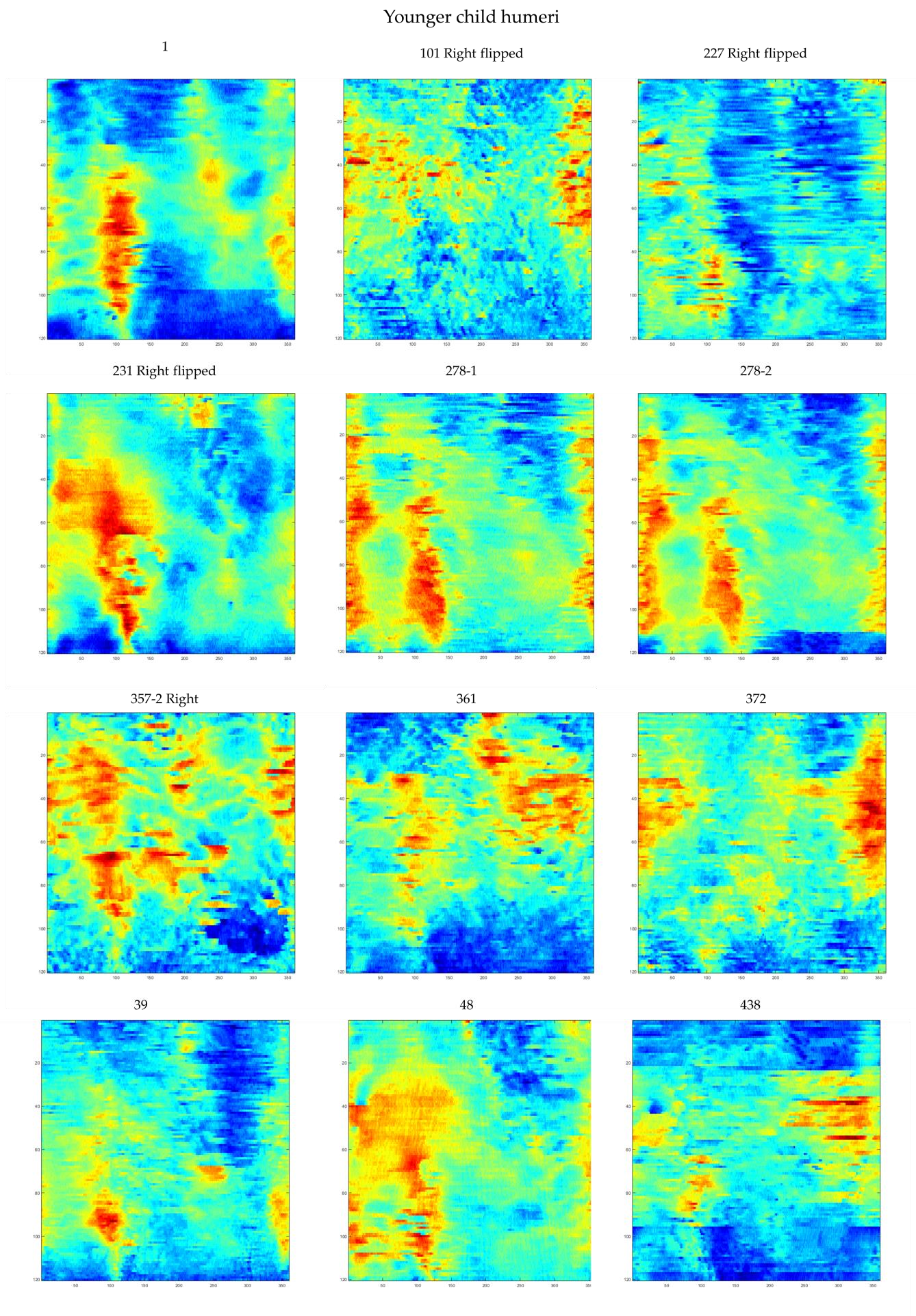


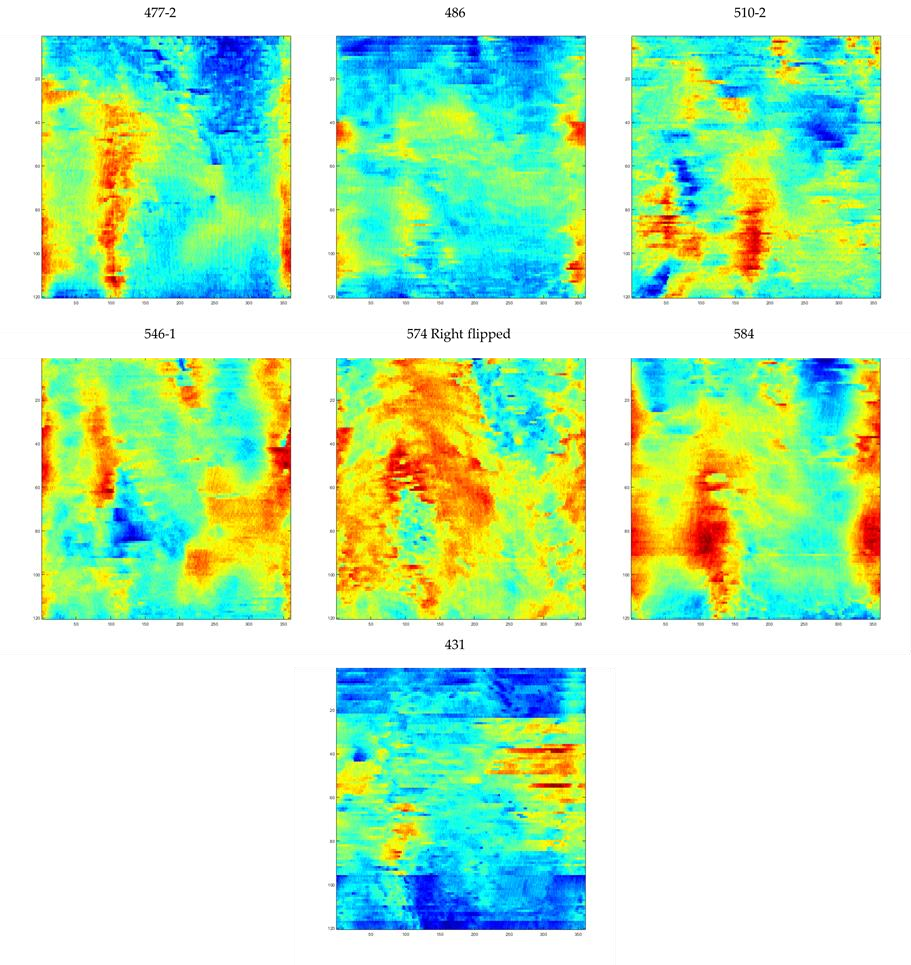


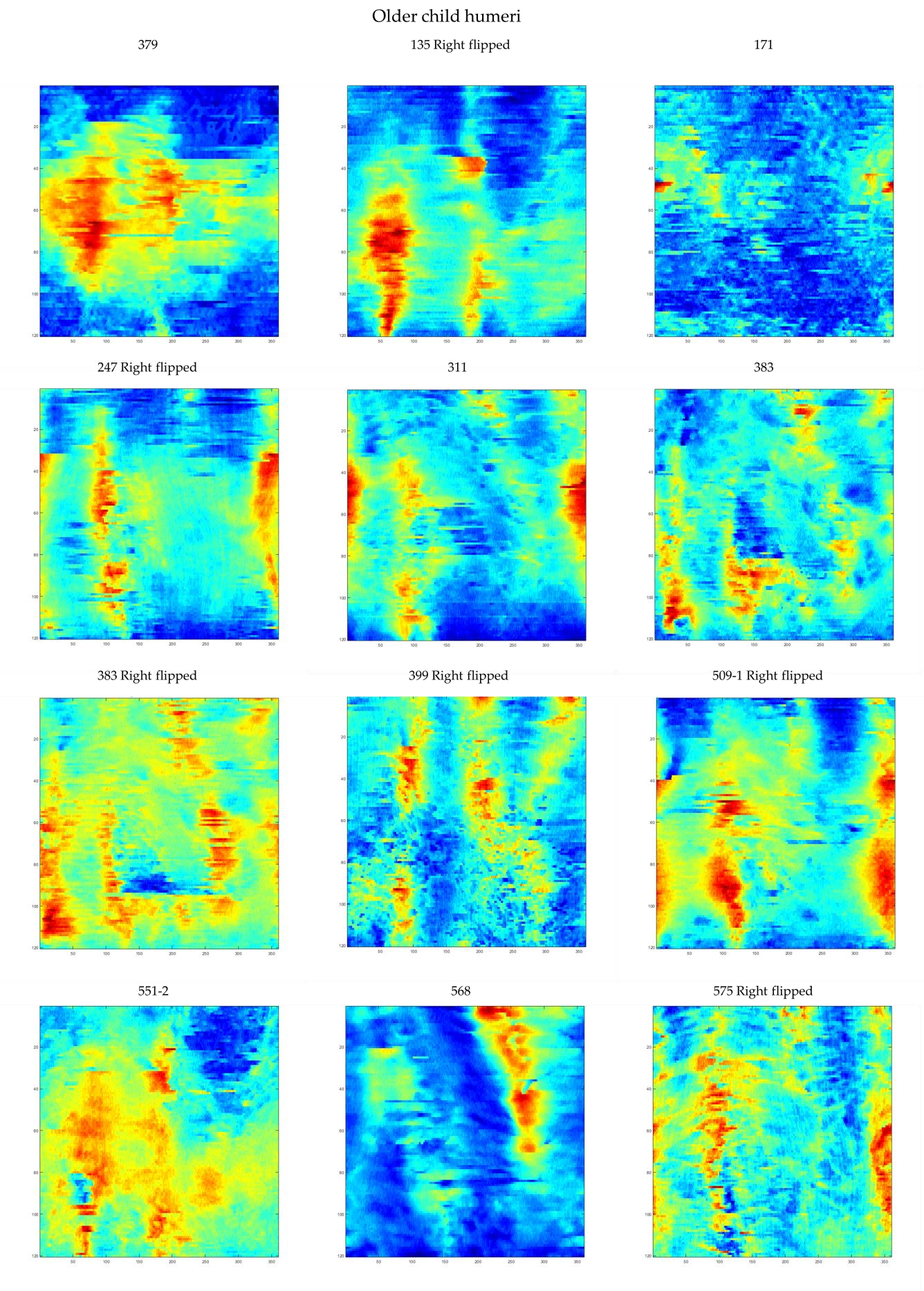


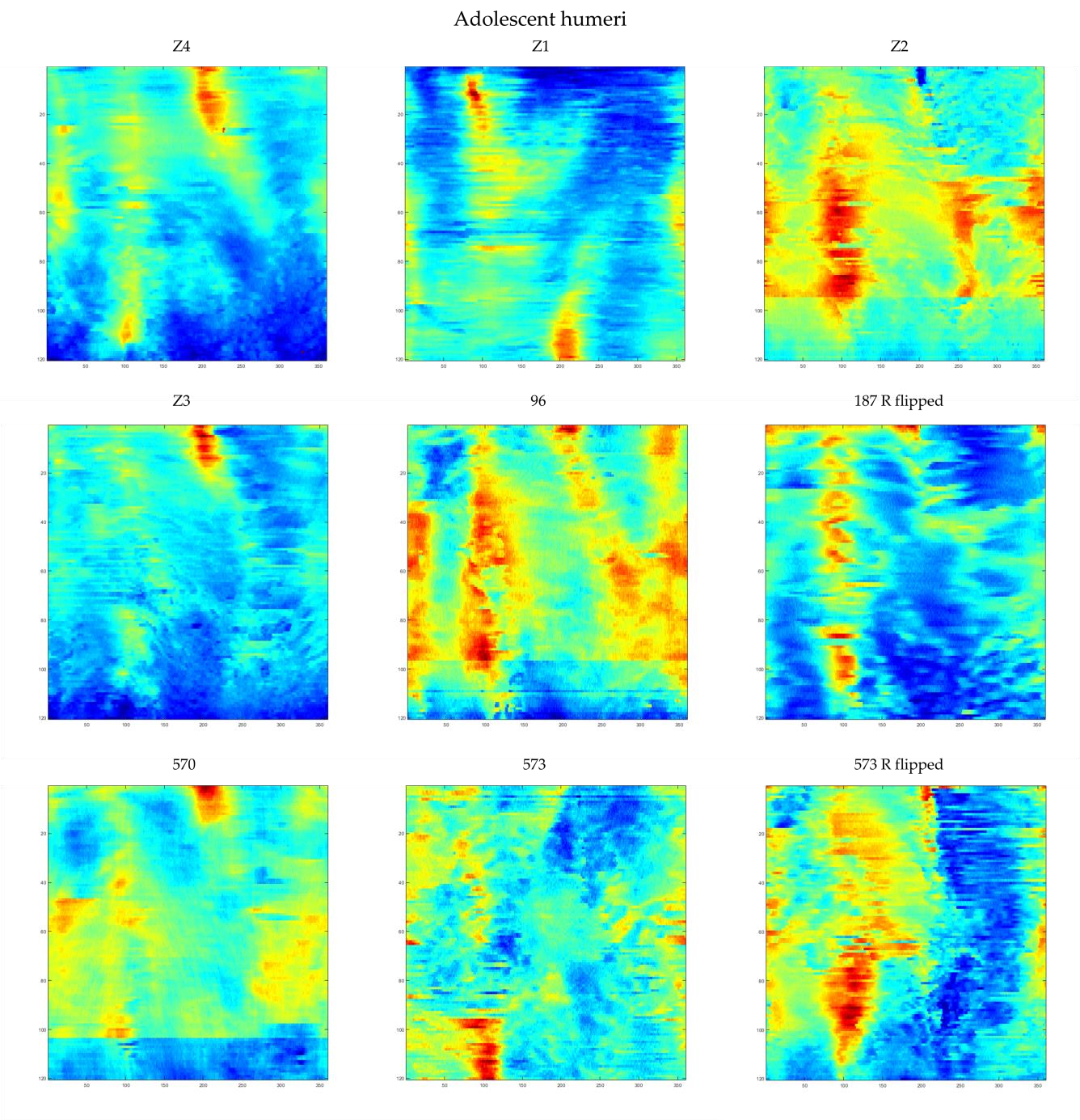


### S3. Periosteal curvature maps for all specimens


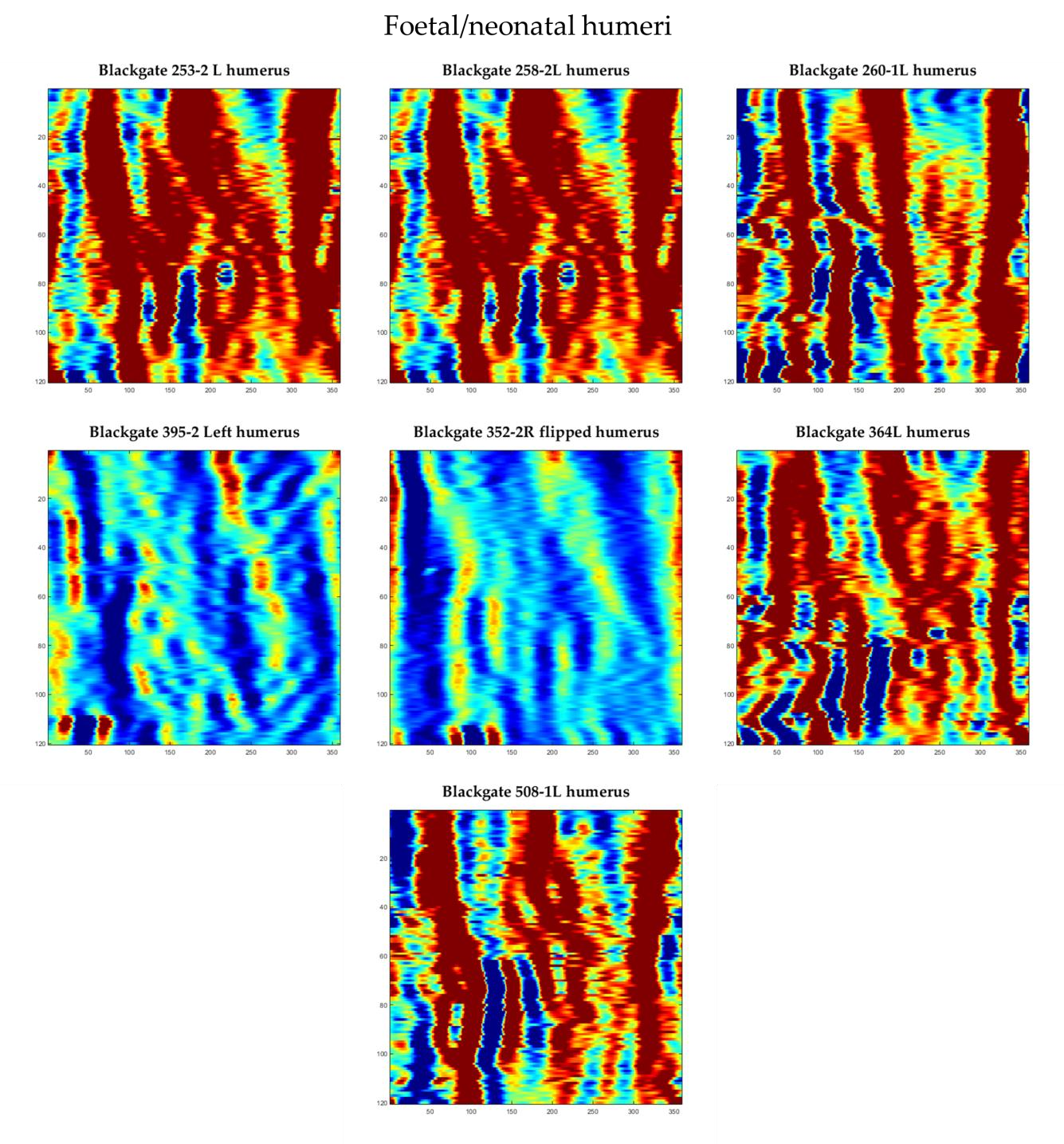


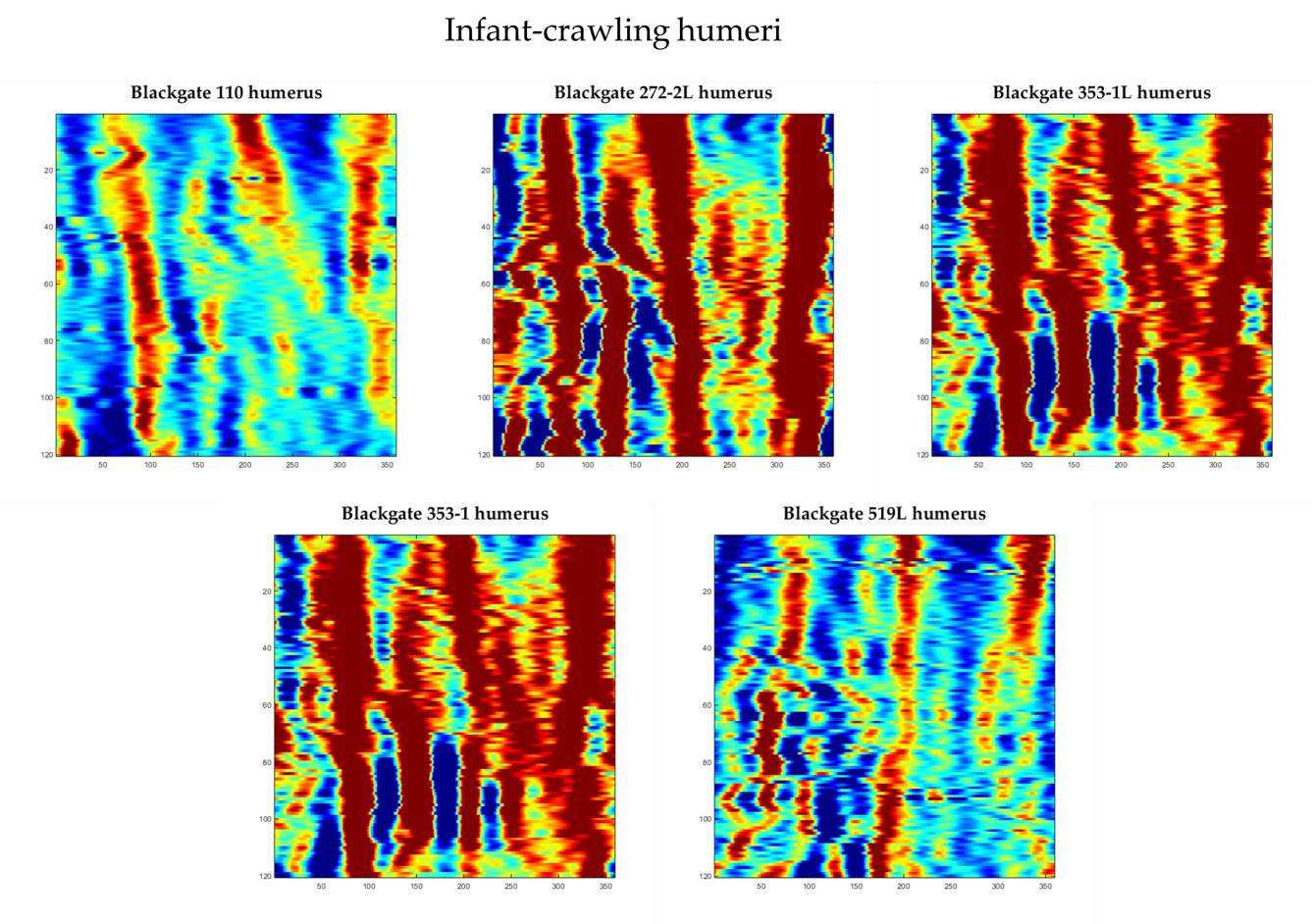


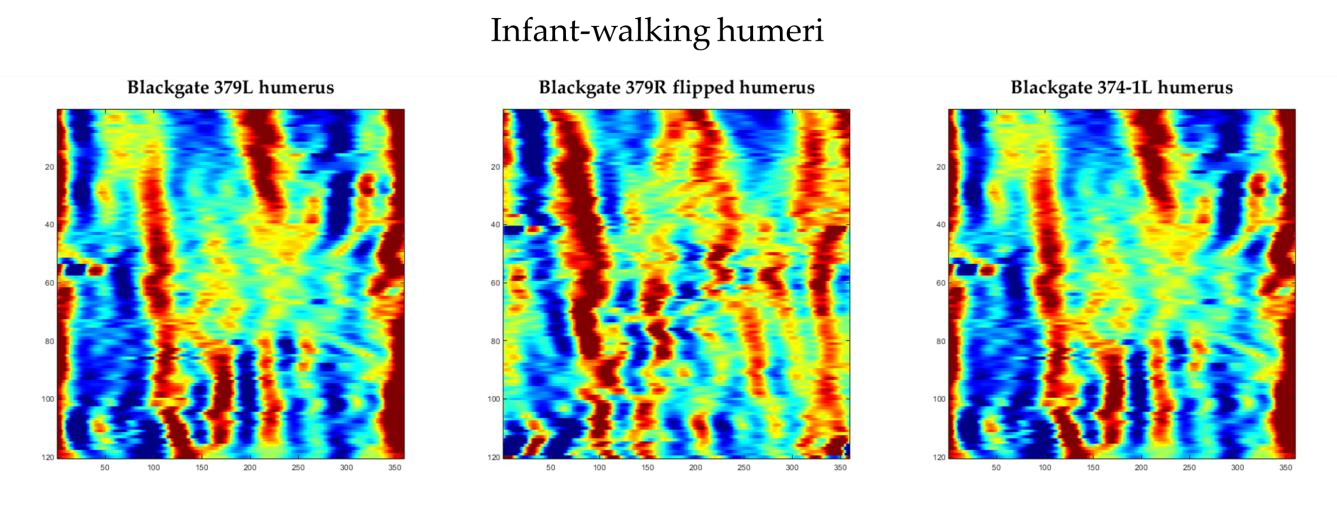


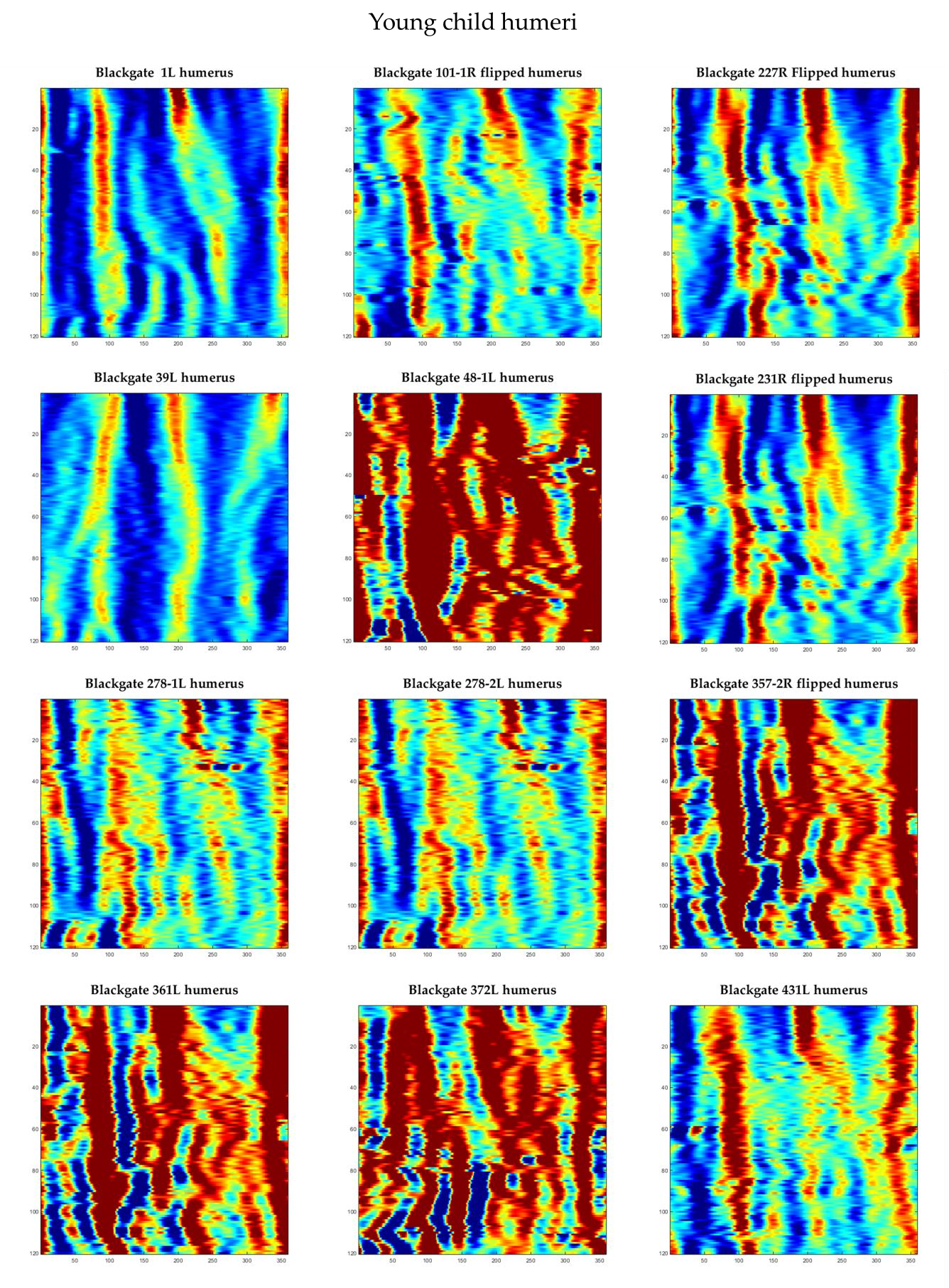


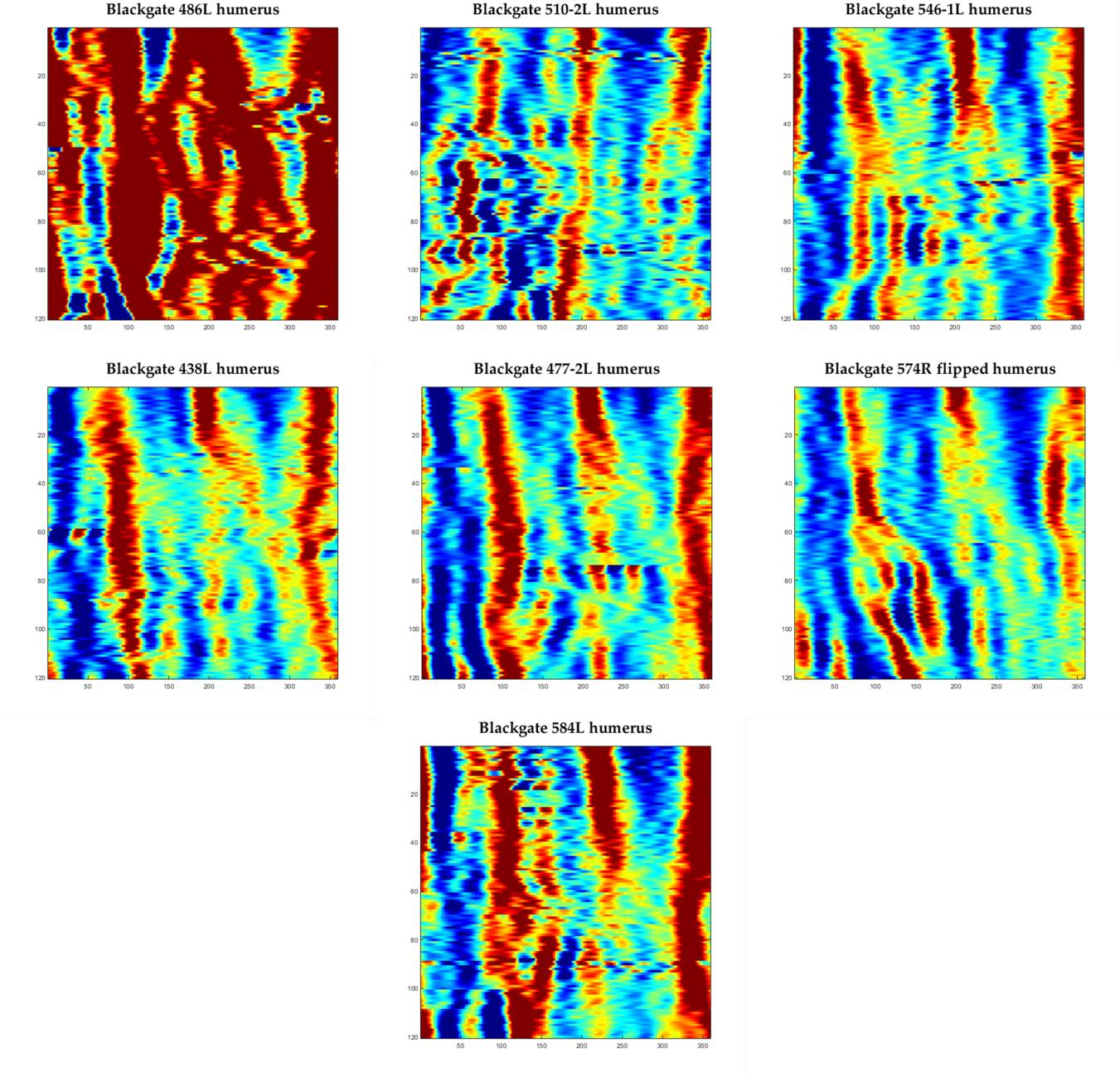


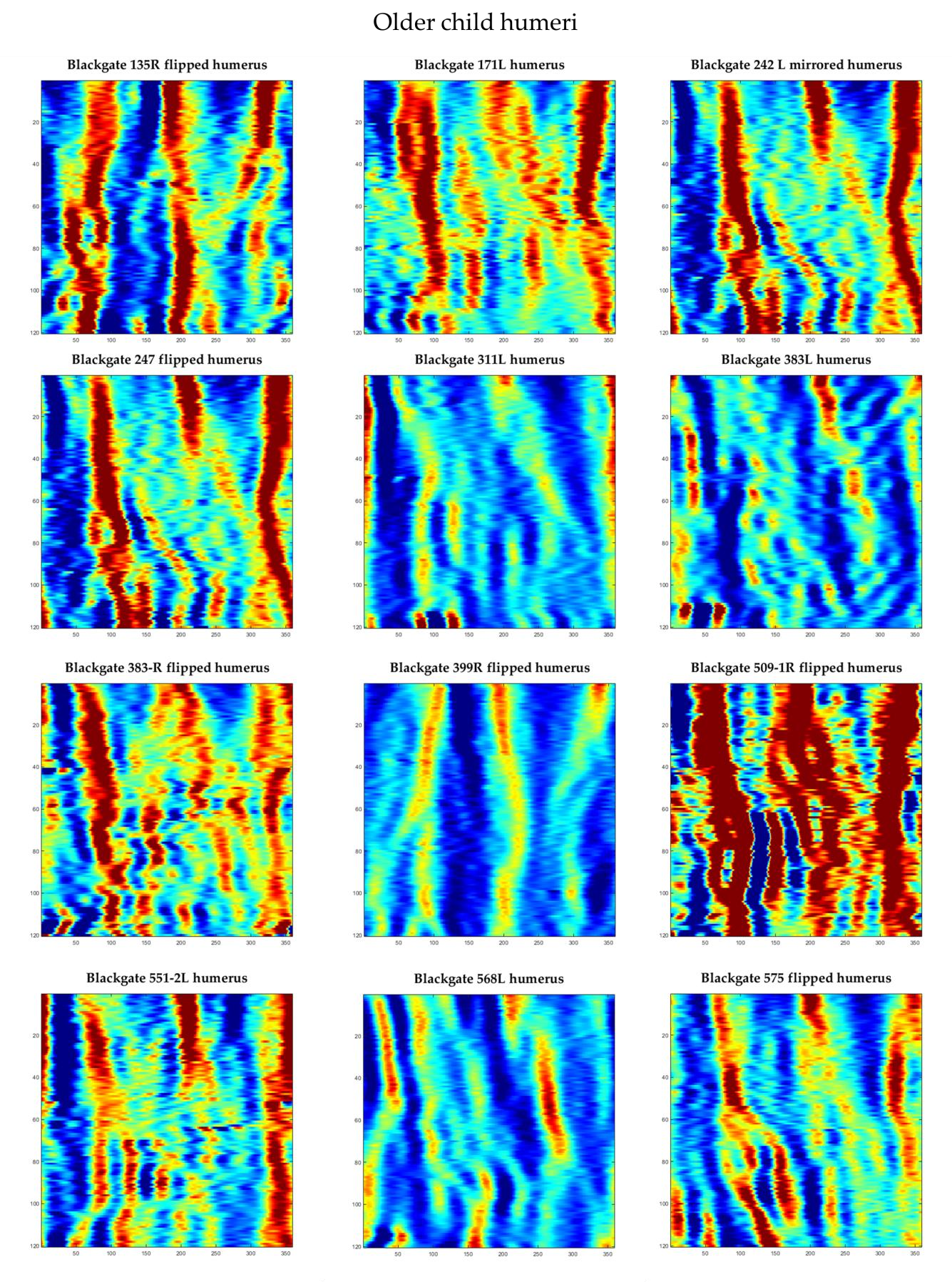


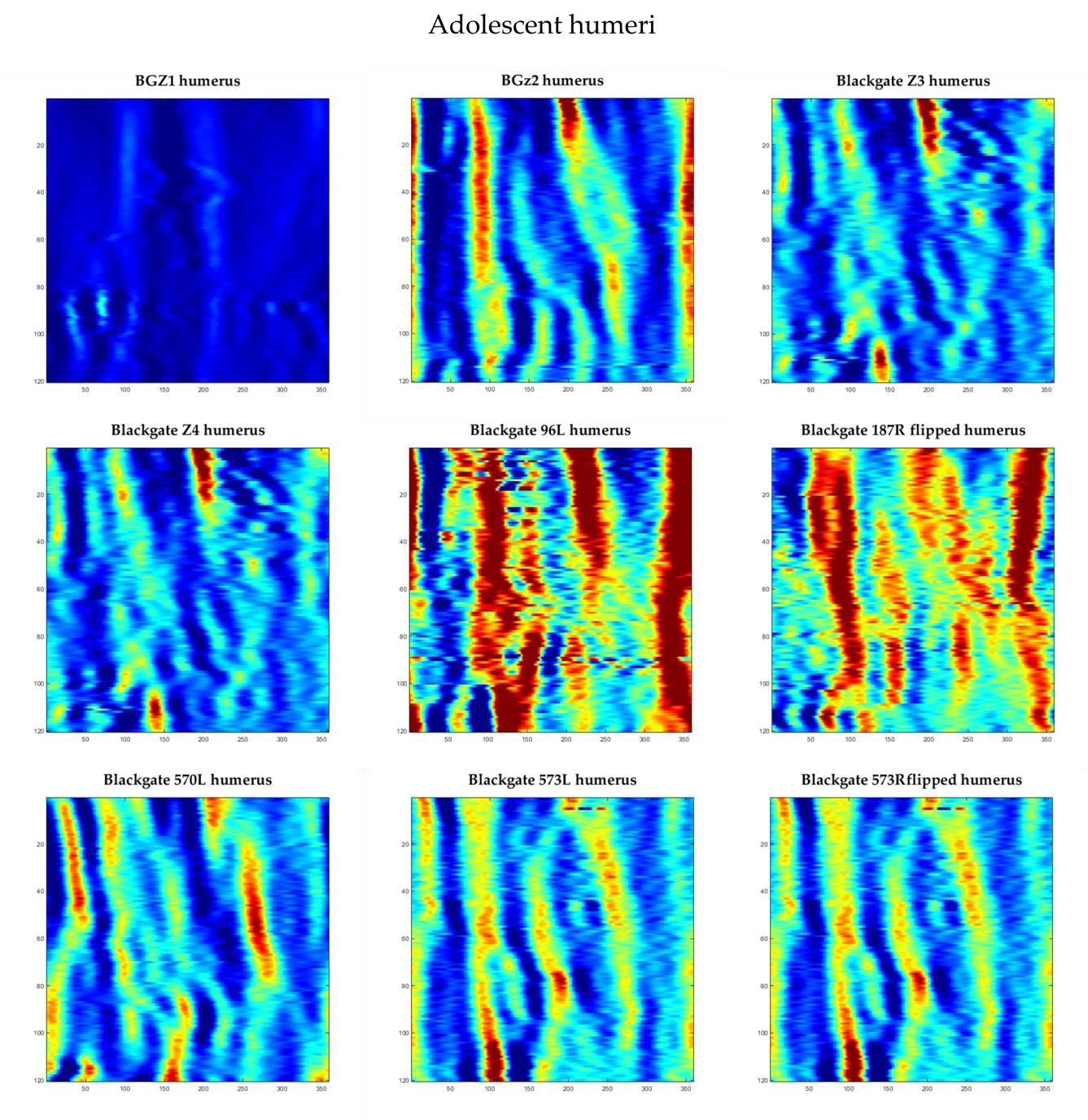


### S4 specimen age estimates and scan parameters

| Specimen no. | Age group | new group | Age Estimate* | Original voxel size | No. of slices | Source to object | Source to detector | Resliced Z res. /mm | Length/mm |
| --- | --- | --- | --- | --- | --- | --- | --- | --- | --- |
| 1 | Young Child | Young Child | 5-6 | 0.086038 | 2014 | 524.4794 | 1198.746 | 0.866402 | 173.3 |
| 39 | Young Child | Infant | 1.5-2.5 | 0.069528 | 1723 | 426.8844 | 1227.944 | 0.598986 | 119.8 |
| 48 | Young Child | Young Child | 5-6 | 0.069528 | 1590 | 426.8844 | 1227.944 | 0.55275 | 110.5 |
| 48 | Young Child | Young Child | 5-6 | 0.069528 | 1590 | 426.8844 | 1227.944 | 0.55275 | 110.5 |
| 96 | Adolescent | Adolescent | 12-15 | 0.0574 | 1462 | 289.1936 | 1007.005 | 0.419594 | 83.9 |
| 96 | Adolescent | Adolescent | 12-15 | 0.0574 | 1462 | 289.1936 | 1007.005 | 0.419594 | 83.9 |
| 101 | Young Child | Infant | 1-1.5 | 0.069528 | 1665 | 426.8844 | 1227.944 | 0.578823 | 115.8 |
| 110 | Infant | Infant | 0.5-1.5 | 0.0673 | 1415 | 356.9686 | 1061.448 | 0.476148 | 95.2 |
| 135 | Older Child | Older Child | 6-8 | 0.097158 | 1688 | 591.4458 | 1217.49 | 0.820015 | 164.0 |
| 141 | Young Child | Young Child | 4.5-5.5 | 0.086038 | 1905 | 507.0948 | 1178.77 | 0.819511 | 163.9 |
| 171 | Infant | Infant | 0.5-1 | 0.108602 | 1269 | 703.7958 | 1296.104 | 0.689078 | 137.8 |
| 171 | Infant | Infant | 0.5-1 | 0.108602 | 1262 | 703.7958 | 1296.104 | 0.685277 | 137.1 |
| 187 | Adolescent | Adolescent | 10-12 | 0.108602 | 2005 | 703.7958 | 1296.104 | 1.088733 | 217.7 |
| 227 | Young Child | Young Child | 2.5-3.5 | 0.080756 | 1632 | 478.9878 | 1186.264 | 0.658967 | 131.8 |
| 227 | Young Child | Young Child | 2.5-3.5 | 0.080756 | 1632 | 478.9878 | 1186.264 | 0.658967 | 131.8 |
| 231 | Young Child | Young Child | 4.5-5.5 | 0.086038 | 1827 | 507.0945 | 1178.771 | 0.785956 | 157.2 |
| 247 | Older Child | Older Child | 8-9 | 0.097158 | 1722 | 591.4458 | 1217.49 | 0.836532 | 167.3 |
| 253 | Foetal/Neonate | Foetal/Neonate | 36-38 weeks I.u | 0.097158 | 1722 | 591.4458 | 1217.49 | 0.836532 | 167.3 |
| 258 | Foetal/Neonate | Foetal/Neonate | 36-38 weeks I.u | 0.0574 | 1312 | 289.1936 | 1007.005 | 0.376544 | 75.3 |
| 258 | Foetal/Neonate | Foetal/Neonate | 36-38 weeks I.u | 0.0574 | 1312 | 289.1936 | 1007.005 | 0.376544 | 75.3 |
| 260 | Foetal/Neonate | Foetal/Neonate | 1-1.25 MO | 0.0574 | 1312 | 289.1936 | 1007.005 | 0.376544 | 75.3 |
| 272 | Foetal/Neonate | Infant | 3-6 MO | 0.051958 | 1350 | 261.6095 | 1007.003 | 0.350717 | 70.1 |
| 278 | Young Child | Young Child | 5-6 | 0.086038 | 1881 | 507.0945 | 1178.771 | 0.809186 | 161.8 |
| 278 | Young Child | Young Child | 5-6 | 0.086038 | 1881 | 507.0945 | 1178.771 | 0.809186 | 161.8 |
| 311 | Older Child | Older Child | 9.5-12.5 | 0.108602 | 2105 | 703.7958 | 1296.104 | 1.143034 | 228.6 |
| 352 | Foetal/Neonate | Foetal/Neonate | 32-34 weeks I.u | 0.0384 | 1346 | 194.01 | 1007.005 | 0.258432 | 51.7 |
| 353 | Foetal/Neonate | Infant | 3-6 MO | 0.0574 | 1439 | 289.1936 | 1007.005 | 0.412993 | 82.6 |
| 353 | Foetal/Neonate | Infant | 3-6 MO | 0.0574 | 1439 | 289.1936 | 1007.005 | 0.412993 | 82.6 |
| 357 | Foetal/Neonate | Foetal/Neonate | 0-1 MO | 0.044172 | 1468 | 228.4256 | 1034.253 | 0.324223 | 64.8 |
| 361 | Young Child | Young Child | 2.5-3.5 | 0.080756 | 1560 | 478.9878 | 1186.264 | 0.629894 | 126.0 |
| 364 | Foetal/Neonate | Foetal/Neonate | 0-2 MO | 0.044172 | 1603 | 228.4256 | 1034.253 | 0.354039 | 70.8 |
| 372 | Young Child | Infant | 1-1.5 | 0.064069 | 1710 | 359.8926 | 1123.446 | 0.547793 | 109.6 |
| 374 | Infant | Foetal/Neonate | 0-1 MO | 0.086038 | 1701 | 507.0948 | 1178.77 | 0.731753 | 146.4 |
| 374 | Infant | Foetal/Neonate | 0-1 month | 0.086038 | 1701 | 507.0948 | 1178.77 | 0.731753 | 146.4 |
| 379 | Infant | Infant | 6-9 MO | 0.069528 | 1351 | 426.8844 | 1227.944 | 0.469664 | 93.9 |
| 383 | Older Child | Older Child | 8.5-9.5 | 0.108602 | 1986 | 703.7958 | 1296.104 | 1.078416 | 215.7 |
| 383 | Older Child | Older Child | 8.5-9.5 | 0.108602 | 1986 | 703.7958 | 1296.104 | 1.078416 | 215.7 |
| 384 | Older Child | Older Child | 7-8 | 0.108602 | 1690 | 703.7982 | 1296.102 | 0.917689 | 183.5 |
| 388 | Young Child | Young Child | 4-5 | 0.086038 | 1779 | 507.0945 | 1178.771 | 0.765307 | 153.1 |
| 395 | Foetal/Neonate | Foetal/Neonate | 0-1 MO | 0.044172 | 1512 | 228.4256 | 1034.253 | 0.333941 | 66.8 |
| 399 | Older Child | Older Child | 11-12 | 0.108602 | 2131 | 703.7958 | 1296.104 | 1.157152 | 231.4 |
| 406 | Young Child | Infant | 1.5-2.5 | 0.080756 | 1434 | 478.9878 | 1186.264 | 0.579018 | 115.8 |
| 431 | Young Child | Young Child | 5-6 | 0.080756 | 1566 | 478.9878 | 1186.264 | 0.632317 | 126.5 |
| 438 | Young Child | Young Child | 5-6 | 0.064069 | 1669 | 359.8926 | 1123.446 | 0.534659 | 106.9 |
| 477 | Young Child | Young Child | 3.5-4.5 | 0.064069 | 1669 | 359.8926 | 1123.446 | 0.534659 | 106.9 |
| 478 | Young Child | Infant | 1-2 | 0.069528 | 1572 | 426.8844 | 1227.944 | 0.546493 | 109.3 |
| 486 | Young Child | Infant | 1.5-2.5 | 0.080756 | 1550 | 478.9878 | 1186.264 | 0.625857 | 125.2 |
| 508 | Foetal/Neonate | Foetal/Neonate | 0-2 MO | 0.051958 | 1317 | 261.6095 | 1007.003 | 0.342144 | 68.4 |
| 509 | Older Child | Older Child | 5.5-6.5 | 0.108602 | 1629 | 703.7982 | 1296.102 | 1.841351 | 176.9 |
| 510 | Young Child | Young Child | 5-6 | 0.108602 | 1725 | 703.7982 | 1296.102 | 0.936694 | 187.3 |
| 519 | Infant | Infant | 1-3 MO | 0.051958 | 1388 | 261.6095 | 1007.003 | 0.360589 | 72.1 |
| 546 | Young Child | Young Child | 4.5-5.5 | 0.108602 | 1611 | 703.7982 | 1296.102 | 0.874791 | 175.0 |
| 546 | Young Child | Young Child | 4.5-5.5 | 0.108602 | 1611 | 703.7982 | 1296.102 | 0.874791 | 175.0 |
| 551 | Infant | Young Child | 3.5-4.5 | 0.108602 | 1611 | 703.7982 | 1296.102 | 0.874791 | 175.0 |
| 568 | Adolescent | Older Child | 9-11 years | 0.108602 | 2111 | 703.7958 | 1296.104 | 1.146292 | 229.3 |
| 570 | Adolescent | Adolescent | 15-17 | 0.108602 | 2111 | 703.7958 | 1296.104 | 1.146292 | 229.3 |
| 573 | Adolescent | Adolescent | 15-17 | 0.108602 | 2111 | 703.7958 | 1296.104 | 1.146292 | 229.3 |
| 573 | Adolescent | Adolescent | 15-17 | 0.108602 | 2111 | 703.7958 | 1296.104 | 1.146292 | 229.3 |
| 574 | Young Child | Young Child | 5-6 | 0.108602 | 1552 | 703.7982 | 1296.102 | 0.842753 | 168.6 |
| 575 | Older Child | Older Child | 11-12 | 0.108602 | 1756 | 703.7982 | 1296.102 | 0.953528 | 190.7 |
| 584 | Young Child | Young Child | 2-3 years | 0.080756 | 1530 | 478.9878 | 1186.264 | 0.617781 | 123.6 |

*Unless otherwise stated-age is in years

### Supplementary figures S5-S7.


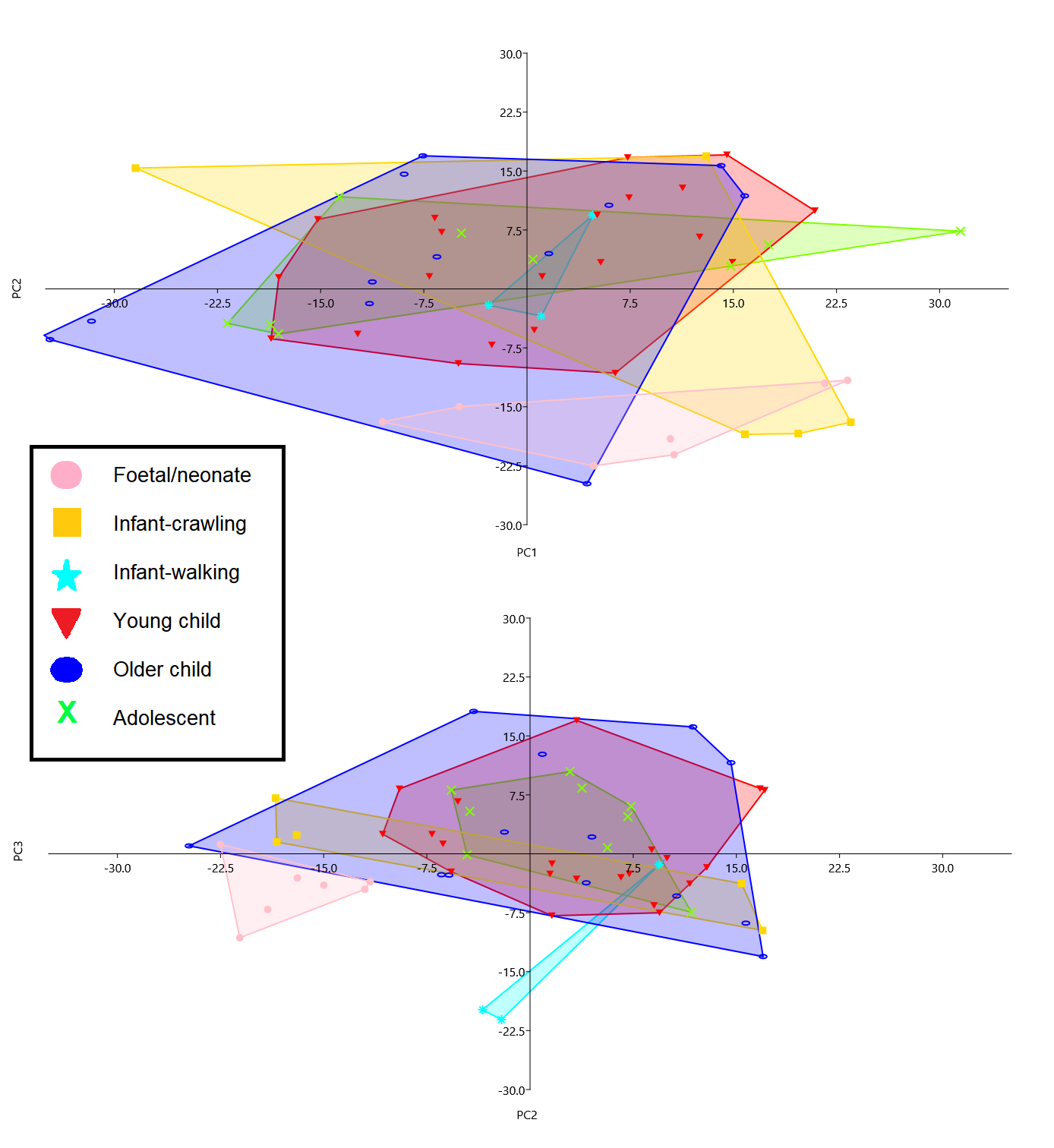


Figure S5. PCA of thickness scores


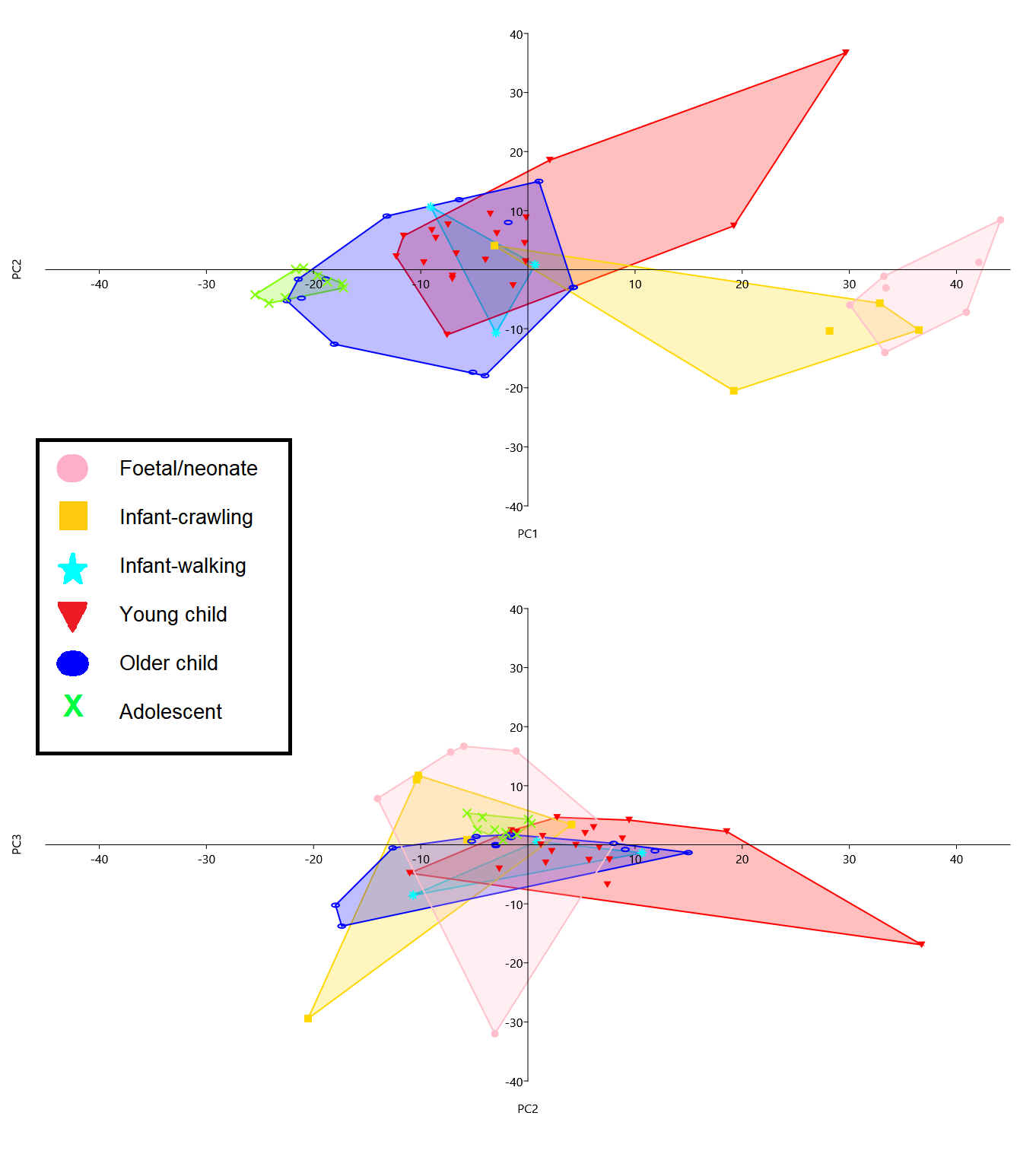


Figure S6. PCA of periosteal curvature scores


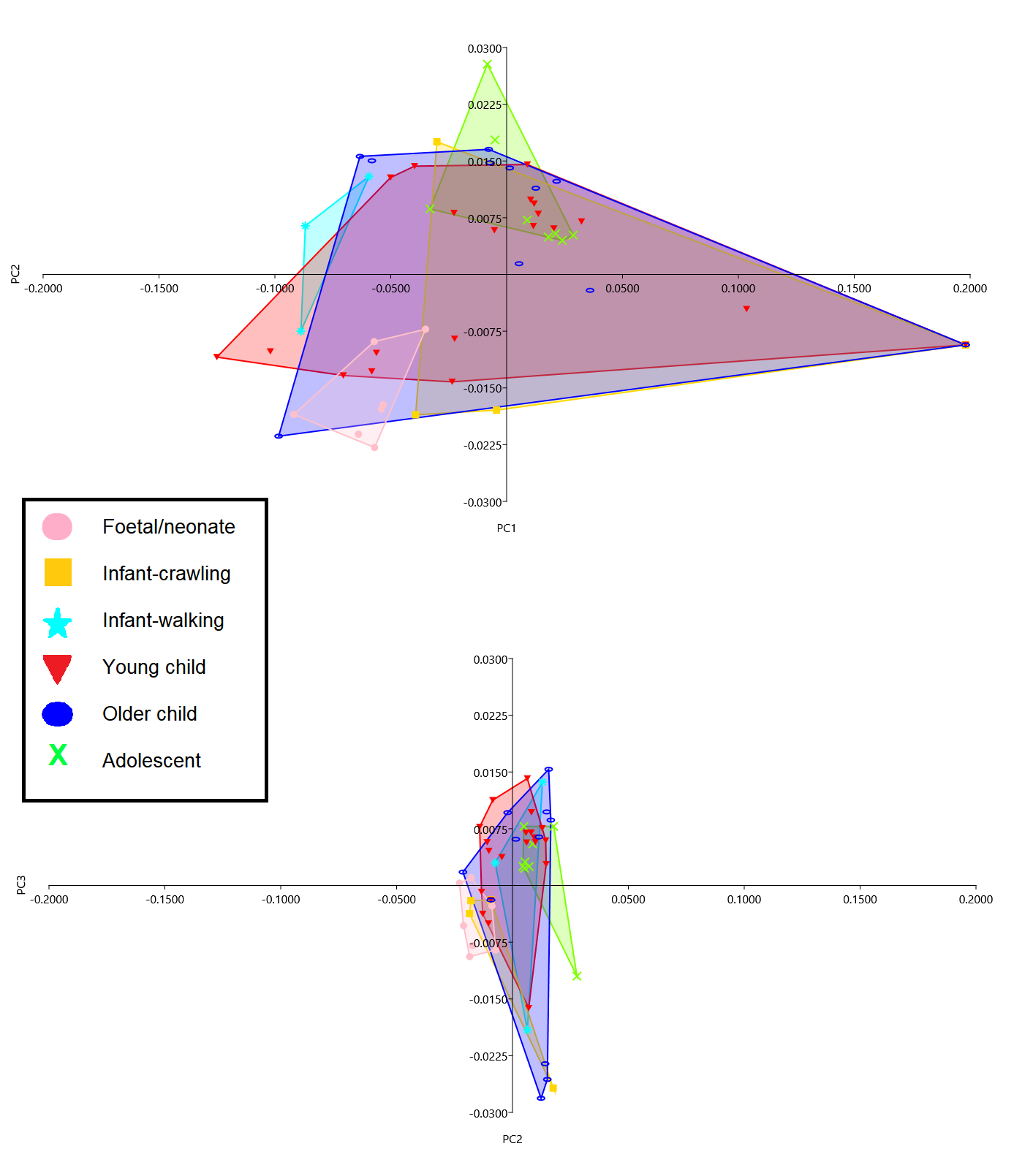


Figure S7. PCA of GMM coordinates

### S8. MATLAB code for thickness calculations

function calculate_thickness_map

% This function looks at all the .tif files in a folder and calculates a thickness map

% it assumes that each tiff file is part of an image stack

num_samples = 360; % this is the number of samples per revolution, so 360 would give a 1 degree interval

slice_subsample = 6; % this is the subsample factor for the innerpoint and outerpoint files sample_subsample = 90; % this is the subsample factor for each slice for the innerpoint and outerpoint files

% first get a folder from the user

folder_name = uigetdir('', 'Choose the folder that contains the image stack'); if folder_name == 0 error('No folder selected');

end

%

%save_name = uigetdir('', 'Choose the folder to save results into');

%if save_name == 0

%error('No folder selected');

%end

% Change the code below to the directory you want to save the results to if ~exist(['C:\Users\~’],'dir') mkdir(['C:\Users\~']) end

save_name = 'C:\Users\mqbpkto2\Dropbox (The University of Manchester)\Thesis chapters\thickness mapping\JULY18'

file_list = dir(fullfile(folder_name,'*.tif')); if isempty(file_list)

error('No .tif files found in folder')

end

%this code will automatically read a csv with metadata in. Change the columns below to %reflect where your voxel sizes are

excel_file_list = dir(fullfile(folder_name,'*metadata.csv')); if isempty(excel_file_list) error('No .csv files found in folder')

end

for file_number = 1:length(excel_file_list) file_name = [folder_name '/' excel_file_list(file_number).name]; metadata = xlsread(file_name);

end mm_per_pixel = metadata(2) slice_thickness = metadata(3)

%This dialogue lets you tell Matlab the dimensions of your file.

%response = inputdlg({'X-Y resolution in mm','Z resolution in mm'}, 'Image Resolution', [1 50; 1

50]);

%if isempty(response)

% error('The size of each image pixel must be specified')

%end

%mm_per_pixel = str2double(response{1});

%slice_thickness = str2double(response{2});

%this dialogue lets you input the start and end percentage for your sequence. %This means that you can just leave all your images in the folder, and not %worry about moving the unwanted slices to another folder.

%responseb = inputdlg({'Start %','end %'}, 'Image Start and End Percent', [1 50; 1 50]);

%if isempty(responseb)

%error('The start and end Percentage must be specified')

%end

%start_image = str2double(responseb{1});

%end_image = str2double(responseb{2});

% or you can alter this code below, if you are going to be doing the same set of measurements repeatedly start_image = 40; end_image = 160; start_perc = start_image;

end_perc = end_image;

%start_image = round((length(file_list)/100)*(str2double(responseb{1})));

%end_image = round((length(file_list)/100)*(str2double(responseb{2})));

%start_image = (round((length(file_list)/100)*(start_perc))); %end_image = round((length(file_list)/100)*(end_perc)); file_numbers = end_image - start_image; file_list_subsample = file_list(start_image:end_image); if ~exist([save_name '\outerpoints\'],'dir') mkdir([save_name '\outerpoints\']) end

if ~exist([save_name '\innerpoints\'],'dir') mkdir([save_name '\innerpoints\']) end

if ~exist([save_name '\slice_geometry\'],'dir') mkdir([save_name '\slice_geometry\']) end

if ~exist([save_name '\outerpoints_subsample\'],'dir') mkdir([save_name '\outerpoints_subsample\']) end

if ~exist([save_name '\thickness\'],'dir') mkdir([save_name '\thickness\']) end

if ~exist([save_name '\innerpoints_subsample\'],'dir') mkdir([save_name '\innerpoints_subsample\']) end

if ~exist([save_name '\thickness_plots\'],'dir') mkdir([save_name '\thickness_plots\']) end

if ~exist([save_name '\thickness_images\'],'dir') mkdir([save_name '\thickness_images\']) end

if ~exist([save_name '\J\'],'dir') mkdir([save_name '\J\']) end

if ~exist([save_name '\CA\'],'dir') mkdir([save_name '\CA\']) end

if ~exist([save_name '\Ix_Iy\'],'dir') mkdir([save_name '\Ix_Iy\']) end

if ~exist([save_name '\Imax_Imin\'],'dir') mkdir([save_name '\Imax_Imin\']) end

%thickness_image = zeros(num_samples,(length(end_image-start_image))) ;

thickness_image = zeros(file_numbers, num_samples);

[pathstr, name, ext] = fileparts(folder_name);

outerpoints_file = fopen([save_name '\outerpoints\' name '_outerpoints.csv'], 'w'); innerpoints_file = fopen([save_name '\innerpoints\' name '_innerpoints.csv'], 'w'); outerpoints_subsample_file = fopen([save_name '\outerpoints_subsample\' name

'_outerpoints_subsample.csv'], 'w');

innerpoints_subsample_file = fopen([save_name '\innerpoints_subsample\' name

'_innerpoints_subsample.csv'], 'w');

J_file = fopen([save_name '\J\' name '_J.csv'], 'w');

CA_file = fopen([save_name '\CA\' name '_CA.csv'], 'w');

Ix_Iy_file = fopen([save_name '\Ix_Iy\' name '_Ix_Iy.csv'], 'w');

Imax_Imin_file = fopen([save_name '\Imax_Imin\' name '_Imax_Imin.csv'], 'w'); centroids_file = fopen([save_name '\slice_geometry\' name '_slice_geometry.csv'], 'w'); fprintf(centroids_file, 'slice, centroid_x, centroid_y, min_thickness, max_thickness, TA, Cortical area, Trabecular area, J, Ix/Iy, Imax/Imin\n', 'w');

fprintf(J_file, 'slice, J\n', 'w'); fprintf(CA_file, 'slice, CA\n', 'w'); fprintf(Ix_Iy_file, 'slice, Ix/Iy\n', 'w');

fprintf(Imax_Imin_file, 'slice, Imax/Imin\n', 'w');

%thickness_file = fopen([save_name '\thickness\' name '_thickness.csv'], 'w');

for slice_counter = 1: length(file_list_subsample);

current_file = fullfile(folder_name,file_list_subsample(slice_counter).name); fprintf('%s\n', current_file);

[thickness, thickness_start, thickness_end, centroid_x, centroid_y] = calculate_thickness(current_file, num_samples); thickness = thickness .* mm_per_pixel; thickness_start = thickness_start .* mm_per_pixel; thickness_end = thickness_end .* mm_per_pixel; centroid_x = centroid_x .* mm_per_pixel; centroid_y = centroid_y .* mm_per_pixel; max_thickness = max(thickness);

min_thickness = min(thickness);

%these following formulae calculate conventional cross sectional properties. TotalA = (polyarea(thickness_end(1:1,1:360),thickness_end(2:2,1:360)));

TrabA = (polyarea(thickness_start(1:1,1:360),thickness_start(2:2,1:360)));

Cortical_area = TotalA-TrabA;

%find Imax distances = sqrt((thickness_end(1:1,1:360)-centroid_x).^2)+((thickness_end(2:2,1:360)centroid_y).^2);

[maxRadius, maxRadiusIndex] = max(distances); maxPointCoord = thickness_end(:,maxRadiusIndex); maxPointCoordinner = thickness_start(:,maxRadiusIndex); opp_point = maxRadiusIndex+180;

if opp_point>360 opp_point = opp_point-360

else

opp_point = opp_point

end

opp_pointCoord = thickness_end(:,opp_point); opp_pointCoordinner = thickness_start(:,opp_point); %extract Imax

Imax = sqrt((((maxPointCoord(1))-(opp_pointCoord(1))).^2)+(((((maxPointCoord(2))(opp_pointCoord(2))).^2))));

Imax_inner = sqrt((((maxPointCoordinner(1))-

(opp_pointCoordinner(1))).^2)+(((((maxPointCoordinner(2))-(opp_pointCoordinner(2))).^2)))); %find Imin

[minRadius, minRadiusIndex] = min(distances); minPointCoord = thickness_end(:,minRadiusIndex); minPointCoordinner = thickness_start(:,minRadiusIndex); opp_pointmin = minRadiusIndex+180;

if opp_pointmin>360

opp_pointmin = opp_pointmin-360 else

opp_pointmin = opp_pointmin end

opp_pointminCoord = thickness_end(:,opp_pointmin); opp_pointminCoordinner = thickness_start(:,opp_pointmin);

%xtract Imin

Imin = sqrt(((minPointCoord(1))-(opp_pointminCoord(1))).^2)+(((((minPointCoord(2))-

(opp_pointminCoord(2))).^2)));

Imin_inner = sqrt(((minPointCoordinner(1))-

(opp_pointminCoordinner(1))).^2)+(((((minPointCoordinner(2))-

(opp_pointminCoordinner(2))).^2)));

%extract max and min second moments of area

Second_moment_of_area_min = (pi*((Imin^4)-(Imin_inner^4)))/32

Second_moment_of_area_max = (pi*((Imax^4)-(Imax_inner^4)))/32

%extractImax/Imin for circularity measure

Ix_Iy = Imax/Imin;

%extract J

J = Second_moment_of_area_min+Second_moment_of_area_max;

%Extract moments of area divided

Imax_Imin = Second_moment_of_area_max/Second_moment_of_area_min;

fprintf('Max thickness = %f\n', max_thickness); fprintf('Min thickness = %f\n', min_thickness);

%fprintf('TA = %f\n', TotalA);

%fprintf('Cortical area = %f\n', Cortical_area);

%fprintf('Trabecular area = %f\n', TrabA);

%fprintf('J = %f\n', J);

%fprintf('Ix/Iy = %f\n', Ix_Iy);

%fprintf('Imax/Imin = %f\n', Imax_Imin);

fprintf(centroids_file, '%d,%g,%g,%g,%g,%g,%g,%g,%g,%g,%g\n', slice_counter, centroid_x, centroid_y, min_thickness, max_thickness, TotalA, Cortical_area, TrabA, J, Ix_Iy, Imax_Imin); fprintf(J_file, '%d, %g\n', slice_counter, J);

fprintf(CA_file, '%d, %g\n', slice_counter, Cortical_area); fprintf(Ix_Iy_file, '%d, %g\n', slice_counter, Ix_Iy);

fprintf(Imax_Imin_file, '%d, %g\n', slice_counter, Imax_Imin);

thickness_image(slice_counter, :) = thickness;

for sample_counter = 1: length(thickness_start);

fprintf(innerpoints_file, '%d,%g,%g\n', (slice_counter*slice_thickness), thickness_start(1, sample_counter), thickness_start(2, sample_counter));

fprintf(outerpoints_file, '%d,%g,%g\n', (slice_counter*slice_thickness), thickness_end(1, sample_counter), thickness_end(2, sample_counter));

%fprintf(thickness_file, thickness);

if (mod(slice_counter, slice_subsample) == 0 && mod(sample_counter, sample_subsample) ==

0)

fprintf(innerpoints_subsample_file, '%d,%g,%g\n', slice_counter.*slice_thickness, thickness_start(1), thickness_start(2));

fprintf(outerpoints_subsample_file, '%d,%g,%g\n', slice_counter.*slice_thickness, thickness_end(1), thickness_end(2)); end

end

end

fclose(outerpoints_file); fclose(innerpoints_file); fclose(outerpoints_subsample_file); fclose(innerpoints_subsample_file);

fclose(centroids_file); fclose(CA_file); fclose(J_file); fclose(Ix_Iy_file);

fclose(Imax_Imin_file);

%thickness_image2 = thickness_image(start_image:end);

% write the thickness data to a CSV file

%csvwrite((fullfile([folder_name] 'thickness_output.csv'), thickness_image);

%[pathstr, name, ext] = fileparts(folder_name);

%csvwrite([save_name '\thickness\' name 'thickness2.csv'], thickness_image2);

%csvwrite([save_name '\thickness\' name 'thickness.csv'], thickness_image);

% produce a suitable image of the thickness map

graph_title1 = ('Cortical thickness of '); graph_title2 = (' humerus. Colour scale is in mm'); graph_bone = (excel_file_list.name(1:end-12)); %graph_bone2 = graph_bone(1:end-12);

graph_title = [graph_title1, graph_bone, graph_title2]; [pathstr, name, ext] = fileparts(folder_name); figure(1);

clf('reset'); set(gcf, 'Color', 'w'); set(gcf,'Units','pixels'); set(gcf, 'Position', [50, 50, 1000, 1000]);

set(gcf,'PaperType','<custom>'); set(gcf,'Units','inches'); set(gcf,'PaperUnits','inches'); position = get(gcf,'Position'); set(gcf,'PaperPosition',[0,0,position(3:4)]); set(gcf,'PaperSize',position(3:4)); imagesc(thickness_image); colormap(jet(256));

%axes1 = axes('Parent',figure(1),...

%'XTickLabel',{'Anterior','Medial','Posterior','Lateral','Anterior'},...

% 'XTick',[0 90 180 270 360],...

%'Layer','top',...

%'YDir','reverse',...

%'YTick',zeros(1,0));

%% Uncomment the following line to preserve the X-limits of the axes

% xlim(axes1,[0.5 360.5]);

%% Uncomment the following line to preserve the Y-limits of the axes

% ylim(axes1,[0.5 4.5]);

%box(axes1,'on');

%hold(axes1,'on');

% Create image

%image(cdata1,'Parent',axes1,'CDataMapping','scaled');

% Create xlabel

xlabel('Bone Position');

% Create ylabel

ylabel({'Distal to proximal humerus.'});

% Create title title(graph_title);

% Create colorbar colorbar('EastOutside'); % Create arrow annotation('arrow',[0.0613750000000001 0.0593750000000001],...

[0.922330097087376 0.107142857142857]); set(gca, 'XTick',[0 90 180 270 360]);

'XTickLabel',{'Anterior','Medial','Posterior','Lateral','Anterior'};,...

plot_name = fullfile(folder_name, 'thickness_plot');

print(gcf,'-dpdf',[save_name '\thickness_plots\' name 'thickness plot', '.pdf'],'-r 150') print(gcf,'-depsc2',[save_name '\thickness_plots\' name 'thickness plot', '.eps'],'-r 150') print(gcf,'-dtiff',[save_name '\thickness_plots\' name 'thickness plot', '.png'],'-r 72') figure(2);

clf('reset'); set(gcf, 'Color', 'w'); set(gcf,'Units','pixels'); set(gcf, 'Position', [50, 50, 1000, 1000]);

set(gcf,'PaperType','<custom>'); set(gcf,'Units','inches'); set(gcf,'PaperUnits','inches'); position = get(gcf,'Position'); set(gcf,'PaperPosition',[0,0,position(3:4)]); set(gcf,'PaperSize',position(3:4)); imagesc(thickness_image); colormap(jet(256));

plot_name2 = fullfile(folder_name, 'thickness_image');

print(gcf,'-dpdf',[save_name '\thickness_images\' name 'thickness_image', '.pdf'],'-r 150') print(gcf,'-depsc2',[save_name '\thickness_images\' name 'thickness_image', '.eps'],'-r 150') print(gcf,'-dtiff',[save_name '\thickness_images\' name 'thickness_image', '.png'],'-r 72')

% ask if the thickness file should be saved somewhere %[pathstr, name, ext] = fileparts(folder_name); default_name = [name, '_thickness.csv'];

%[file_name, path_name] = uiputfile('*.csv', 'Save the thickness image', default_name);

%if file_name ~= 0

%csvwrite(fullfile(path_name, file_name), thickness_image); return

function [thickness, thickness_start, thickness_end, centroid_x, centroid_y] = calculate_thickness(current_file, num_samples)

% this function calculates the thickness of the bone wall

% it produces num_samples values from angle zero to 360

% zero angle is aligned with the X axis and increases counterclockwise

% read the image data = imread(current_file); [height, width, channels] = size(data);

% now do some sanity checking on the image

if channels ~= 1

error('Only greyscale images supported'); end

if data(1, 1) ~= data(1, width) || data(1, 1) ~= data(height, width) || data(1, 1) ~= data(height, 1) error('All 4 corners of the image must have the same greyscale value'); end

background = data(1, 1);

% find the centroid xsum = 0; ysum = 0; count = 0; for ix = 1: width for iy = 1: height

if data(iy, ix) ~= background xsum = xsum + ix; ysum = ysum + iy; count = count + 1;

end

end end

if count == 0 % must be a completely blank image so set all the thicknesses to zero fprintf('Centroid not found\n'); thickness = zeros(1, file_numbers); return end

centroid_x = xsum / count; centroid_y = ysum / count;

fprintf('Centroid x = %f y = %f\n', centroid_x, centroid_y);

% now work radially around from centroid

thickness = 9999 * ones(1, num_samples); % create a suitable row vector to hold the thicknesses using dummy big values

thickness_start = zeros(2, num_samples); thickness_end = zeros(2, num_samples); for i = 1: num_samples

circle_fraction = (i - 1) / num_samples; angle = 2 * pi() * circle_fraction; del_x = cos(angle); del_y = sin(angle); % look for the inner edge

for r = 0: 0.5: max([width, height]) % the 0.5 here gets us around any potential rounding error problems

x = r * del_x + centroid_x; y = r * del_y + centroid_y; ix = round(x); iy = round(y);

if (ix < 1 || ix > width || iy < 1 || iy > height)

% if we can't find the inner edge then there is a complete hole here so set the thickness to zero

thickness(i) = 0;

break;

end

if data(iy, ix) ~= background % found the inner edge thickness_start(1, i) = ix; thickness_start(2, i) = iy;

break; end end if thickness(i) ~= 0

% look for the outer edge

for r = max([width, height]): -0.5: 0 % the -0.5 here gets us around any potential rounding error problems

x = r * del_x + centroid_x; y = r * del_y + centroid_y; ix = round(x); iy = round(y);

if (ix < 1 || ix > width || iy < 1 || iy > height)

continue; % this isn't an error, we just haven't found the edge of the image yet end

if data(iy, ix) ~= background % found the outer edge thickness_end(1, i) = ix; thickness_end(2, i) = iy;

break; end end

% if the outer edge is the same as the inner edge it means that the radial line has met at the same pixel

% so in fact the distance between the outer edge and inner edge is always 1 pixel too small so

%we need to correct for this

thickness(i) = 1 + sqrt((thickness_end(1, i) - thickness_start(1, i))^2 + (thickness_end(2, i) - thickness_start(2, i))^2);

end

end

return

### S9. MATLAB code for periosteal curvature calculations

function k_fouriercurvature

%subsample = 10

% read the csv folder containing the outline coordinates for each stack folder_name = uigetdir('', 'Choose the folder that contains the image stack'); if folder_name == 0 error('No folder selected');

end

file_list = dir(fullfile(folder_name,'*.csv')); if isempty(file_list)

error('No .csv files found in folder')

end

%make a subdirectory for each output to be saved in. if ~exist([folder_name '\curvature_rawpoints\'],'dir') mkdir([folder_name '\curvature_rawpoints\']) end

if ~exist([folder_name '\curvature_heatmap\'],'dir') mkdir([folder_name '\curvature_heatmap\']) end

if ~exist([folder_name '\curvature_2dgraph\'],'dir') mkdir([folder_name '\curvature_2dgraph\']) end

if ~exist([folder_name '\curvature_values\'],'dir') mkdir([folder_name '\curvature_values\']) end

if ~exist([folder_name '\smoothed_values\'],'dir') mkdir([folder_name '\smoothed_values\']) end

for file_number = 1:length(file_list)

file_name = [folder_name '/' file_list(file_number).name];

[pathstr, name, ext] = fileparts(file_name);

file_name2 = [folder_name '\curvature_values\' file_list(file_number).name(1:end-4)

'curvature.csv'];

file_name3 = [folder_name '\smoothed_values\' file_list(file_number).name(1:end-4) 'smoothed outline.csv'];

heatmap_name = [folder_name '\curvature_heatmap\' file_list(file_number).name(1:end-4)

'curvature_heatmap'];

smoothoutline_name = [folder_name '\curvature_2dgraph\' file_list(file_number).name(1:end-4)

'curvature_smoothed'];

outline_name = [folder_name '\curvature_rawpoints\' file_list(file_number).name(1:end-4)

'original_curvature']

graph_bone_name = [name(1:end-12)];

fourierpoints = csvread(file_name);

slice_number = fourierpoints(:, 1); x_list = fourierpoints(:, 2);

y_list = fourierpoints(:, 3);

% count the number of points in a slice points_per_slice = 1; for i = 1: length(slice_number) - 1 if (slice_number(i + 1) ~= slice_number(i)) break; end

points_per_slice = points_per_slice + 1; end

slice_number = reshape(slice_number, [points_per_slice, length(slice_number) / points_per_slice]);

% this reshape needs checking

x_list = reshape(x_list, [points_per_slice, length(x_list) / points_per_slice]); y_list = reshape(y_list, [points_per_slice, length(y_list) / points_per_slice]);

[r, c] = size(slice_number); num_slices = c; num_points_wanted = 360;

k_map = zeros(num_slices, num_points_wanted);

l = zeros(num_slices*num_points_wanted,3); for i = 1: 3 figure(i) clf('reset'); set(gcf, 'Color', 'w'); set(gcf,'Units','pixels');

set(gcf, 'Position', [i + 50, i + 50, 1000, 1000]); end

for slice = 1: num_slices

1. = x_list(1:points_per_slice, slice);
2. = y_list(1:points_per_slice, slice);

figure(1); plot(x, y);

hold on;

plot_name3 = outline_name;

title(['Original endosteal contour of ' graph_bone_name ' humerus from a µCT scan']); xlabel('X'); ylabel('Y');

%print(gcf,'-dtiff',[plot_name3, '.png'],'-r 72');

%print({(gcf'-dtiff', plot_name3, '.png')} '-r 72');

%print(gcf,'-depsc2','.eps')

%hold off;

%print(gcf,'-dtiff',[plot_name3, '.png'],'-r 72')

outline = [x, y];

iNoOfHarmonicsAnalyse = points_per_slice / 2;

bNormaliseSizeState = 0; bNormaliseOrientationState = 0;

rFSDs = fEfourier(outline, iNoOfHarmonicsAnalyse, bNormaliseSizeState, bNormaliseOrientationState);

% the degree of smoothing depends on the number of harmonics we choose iNoOfHarmonicsReconstruct = 10; iNoOfPointsReconstruct = num_points_wanted;

smoothed_outln = rEfourier(rFSDs, iNoOfHarmonicsReconstruct, iNoOfPointsReconstruct);

%%%%% Jamie added in bit to output smoothed_oultn variable smoothed_outln_stored(:,2*slice-1) = smoothed_outln(:,1); smoothed_outln_stored(:,2*slice) = smoothed_outln(:,2);

if slice == num_slices

%B = reshape(smoothed_outln_stored,[],2)

csvwrite([file_name(1:end-4) '_smoothed_outln.csv'],smoothed_outln_stored)

%csvwrite([file_name(1:end-4) '_smoothed_outln2.csv'],B) end

if slice == num_slices

B = reshape(smoothed_outln_stored,[],2)

csvwrite([file_name(1:end-4) '_smoothed_outln2.csv'],B) end

%%%%%%%%%%%%%%%%%%%%%%%%%%%%%%%%%%%%%%%%%%%%%%%%%%%%

%%%%%%

%l_map(slice, :) = smoothed_outln; figure(2);

plot(smoothed_outln(:, 1), smoothed_outln(:, 2)); hold on;

k_coeff = calc_k_coefficent(smoothed_outln(:, 1), smoothed_outln(:, 2)); k_map(slice, :) = k_coeff;

title(['Smoothed endosteal contour of ' graph_bone_name ' humerus from a µCT scan']); xlabel('X');

ylabel('Y');

plot_name2 = smoothoutline_name;

%print(gcf,'-dtiff',[plot_name2, '.png'],'-r 72'); end

print(gcf,'-dtiff',[plot_name2, '.png'],'-r 72') print(gcf,'-dtiff',[plot_name3, '.png'],'-r 72') figure(3) clf('reset'); set(gcf, 'Color', 'w'); set(gcf,'Units','pixels');

set(gcf, 'Position', [50, 50, 1000, 1000]); imagesc(k_map);

set(gcf,'PaperType','<custom>'); set(gcf,'Units','inches'); set(gcf,'PaperUnits','inches'); position = get(gcf,'Position'); set(gcf,'PaperPosition',[0,0,position(3:4)]);

set(gcf,'PaperSize',position(3:4)); plot_name = [heatmap_name];

colormap(jet(256)); colorbar('EastOutside'); xlabel('Bone Position'); ylabel({'Slice number.','Lower numbers are more proximal'}); title(['Surface curvature of ' graph_bone_name ' humerus']);

%print(gcf,'-dtiff',[plot_name3, '.png'],'-r 72');

%print(gcf,'-dpdf','Curvature Map.pdf','-r 150')

%print(gcf,'-depsc2','Curvature Map.eps','-r 150')

%print (gcf,'-depsc2','filename2 Curvature Map.eps','-r 150')

print(gcf,'-dpdf',[plot_name, '.pdf'],'-r 300') print(gcf,'-depsc2',[plot_name, '.eps'],'-r 300') print(gcf,'-dtiff',[plot_name, '.png'],'-r 72')

% output the k values per slice to a file

%if (file_name2 ~= 0) fout = fopen((file_name2), 'w'); for slice = 1: num_slices fprintf(fout, '%d', slice); k = k_map(slice, :); for t = 1: length(k) fprintf(fout, ',%f', k(t)); end

fprintf(fout, '\n');

end fclose(fout);

fout2 = fopen((file_name3), 'w');

end

function rFSDs = fEfourier(outline, iNoOfHarmonicsAnalyse, bNormaliseSizeState, bNormaliseOrientationState)

% Forward elliptical Fourier transform - see Kuhl FP and Giardina CR % "Elliptic Fourier features of a closed contour" Computer Graphics and % Image Processing 18:236-258 1982 for theory.

% Returns a shape spectrum of input x,y data "outline" with % iNoOfHarmonicsAnalyse elements.

% The output FSDs will be normalised for location, size and orientation

% if bNormaliseSizeState and bNormaliseOrientationState are TRUE

% Pre-calculate some constant arrays

% n * 2 * pi

% n^2 * 2* pi^2

% where n is the number of harmonics to be used in the analysis rTwoNPi = (1:1:iNoOfHarmonicsAnalyse)* 2 * pi; rTwoNSqPiSq = (1:1:iNoOfHarmonicsAnalyse) .* (1:1:iNoOfHarmonicsAnalyse)* 2 * pi * pi;

iNoOfPoints = size(outline,1) - 1; % hence there is 1 more data point in outline than iNoOfPoints rDeltaX = zeros(iNoOfPoints+1,1); % pre-allocate some arrays rDeltaY = zeros(iNoOfPoints+1,1);

rDeltaT = zeros(iNoOfPoints+1,1);

for iCount = 2 : iNoOfPoints + 1

rDeltaX(iCount-1) = outline(iCount,1) - outline(iCount-1,1); rDeltaY(iCount-1) = outline(iCount,2) - outline(iCount-1,2); end

% Calculate 'time' differences from point to point - actually distances, but we are

% carrying on the fiction of a point running around the closed figure at constant speed.

% We are analysing the projections on to the x and y axes of this point's path around the figure for iCount = 1 : iNoOfPoints

rDeltaT(iCount) = sqrt((rDeltaX(iCount)^2) + (rDeltaY(iCount)^2)); end check = (rDeltaT ~= 0); % remove zeros from rDeltaT, rDeltaX...

rDeltaT = rDeltaT(check); rDeltaX = rDeltaX(check); rDeltaY = rDeltaY(check);

iNoOfPoints = size(rDeltaT,1) - 1; % we have removed duplicate points % now sum the incremental times to get the time at any point rTime(1) = 0;

for iCount = 2 : iNoOfPoints + 1

rTime(iCount) = rTime(iCount - 1) + rDeltaT(iCount-1); end

rPeriod = rTime(iNoOfPoints+1); % rPeriod defined for readability

% calculate the A-sub-0 coefficient rSum1 = 0;

for iP = 2 : iNoOfPoints + 1 rSum2 = 0; rSum3 = 0; rInnerDiff = 0;

% calculate the partial sums - these are 0 for iCount = 1 if iP > 1 for iJ = 2 : iP-1

rSum2 = rSum2 + rDeltaX(iJ-1); rSum3 = rSum3 + rDeltaT(iJ-1); end

rInnerDiff = rSum2 - ((rDeltaX(iP-1) / rDeltaT(iP-1)) * rSum3); end

rIncr1 = ((rDeltaX(iP-1) / (2*rDeltaT(iP-1)))*(rTime(iP)^2-rTime(iP-1)^2) + rInnerDiff*(rTime(iP)rTime(iP-1))); rSum1 = rSum1 + rIncr1; end

rFSDs(1,1) = ((1 / rPeriod) * rSum1) + outline(1,1); % store A-sub-0 in output FSDs array - this array will be 4 x iNoOfHarmonicsAnalyse % calculate the a-sub-n coefficients

for iHNo = 2 : iNoOfHarmonicsAnalyse rSum1 = 0; for iP = 1 : iNoOfPoints

rIncr1 = (rDeltaX(iP) / rDeltaT(iP))*((cos(rTwoNPi(iHNo-1)*rTime(iP+1)/rPeriod) - cos(rTwoNPi(iHNo-1)*rTime(iP)/rPeriod)));

rSum1 = rSum1 + rIncr1;

end

rFSDs(1,iHNo) = (rPeriod / rTwoNSqPiSq(iHNo-1)) * rSum1; end % "foriHNo = 1 :..."

rFSDs(2,1) = 0; % there is no 0th order sine coefficient

% calculate the b-sub-n coefficients

for iHNo = 2 : iNoOfHarmonicsAnalyse rSum1 = 0; for iP = 1 : iNoOfPoints

rIncr1 = (rDeltaX(iP) / rDeltaT(iP))*((sin(rTwoNPi(iHNo-1)*rTime(iP+1)/rPeriod) - sin(rTwoNPi(iHNo-1)*rTime(iP)/rPeriod)));

rSum1 = rSum1 + rIncr1; end

rFSDs(2,iHNo) = (rPeriod / rTwoNSqPiSq(iHNo-1)) * rSum1; end % "foriHNo = 1 :..." % calculate the C-sub-0 coefficient rSum1 = 0;

for iP = 2 : iNoOfPoints + 1 rSum2 = 0; rSum3 = 0; rInnerDiff = 0;

% calculate the partial sums - these are 0 for iCount = 1 if iP > 1 for iJ = 2 : iP-1

rSum2 = rSum2 + rDeltaY(iJ-1); rSum3 = rSum3 + rDeltaT(iJ-1); end

rInnerDiff = rSum2 - ((rDeltaY(iP-1) / rDeltaT(iP-1)) * rSum3); end

rIncr1 = ((rDeltaY(iP-1) / (2*rDeltaT(iP-1)))*(rTime(iP)^2-rTime(iP-1)^2) + rInnerDiff*(rTime(iP)rTime(iP-1))); rSum1 = rSum1 + rIncr1; end

rFSDs(3,1) = ((1 / rPeriod) * rSum1) + outline(1,2); % store C-sub-0 in output FSDs array - this array will be 4 x iNoOfHarmonicsAnalyse

% calculate the C-sub-n coefficients

for iHNo = 2 : iNoOfHarmonicsAnalyse rSum1 = 0; for iP = 1 : iNoOfPoints

rIncr1 = (rDeltaY(iP) / rDeltaT(iP))*((cos(rTwoNPi(iHNo-1)*rTime(iP+1)/rPeriod) - cos(rTwoNPi(iHNo-1)*rTime(iP)/rPeriod)));

rSum1 = rSum1 + rIncr1;

end

rFSDs(3,iHNo) = (rPeriod / rTwoNSqPiSq(iHNo-1)) * rSum1; end % "foriHNo = 1 :..."

rFSDs(4,1) = 0; % there is no 0th order sine coefficient

% calculate the D-sub-n coefficients

for iHNo = 2 : iNoOfHarmonicsAnalyse rSum1 = 0; for iP = 1 : iNoOfPoints

rIncr1 = (rDeltaY(iP) / rDeltaT(iP))*((sin(rTwoNPi(iHNo-1)*rTime(iP+1)/rPeriod) - sin(rTwoNPi(iHNo-1)*rTime(iP)/rPeriod)));

rSum1 = rSum1 + rIncr1;

end

rFSDs(4,iHNo) = (rPeriod / rTwoNSqPiSq(iHNo-1)) * rSum1; end % "foriHNo = 1 :...

% the non-normalised coefficients are now in rFSDs % if we want the normalised ones, this is where it happens if (bNormaliseSizeState == 1) || (bNormaliseOrientationState == 1) % rTheta1 is the angle through which the starting position of the first

% harmonic phasor must be rotated to be aligned with the major axis of

% the first harmonic ellipse rFSDsTemp = rFSDs;

rTheta1 = 0.5 * atan(2 * (rFSDsTemp(1,2) * rFSDsTemp(2,2) + rFSDsTemp(3,2) * rFSDsTemp(4,2)) /

...

(rFSDsTemp(1,2)^2 + rFSDsTemp(3,2)^2 - rFSDsTemp(2,2)^2 - rFSDsTemp(4,2)^2));

% calculate the partially normalised coefficients - normalised for

% starting point

for iHNo = 1 : iNoOfHarmonicsAnalyse

rStarFSDs(1,iHNo) = cos((iHNo-1) * rTheta1) * rFSDsTemp(1,iHNo) + sin((iHNo-1) * rTheta1) * rFSDsTemp(2,iHNo);

rStarFSDs(2,iHNo) = -sin((iHNo-1) * rTheta1) * rFSDsTemp(1,iHNo) + cos((iHNo-1) * rTheta1)

- rFSDsTemp(2,iHNo);

rStarFSDs(3,iHNo) = cos((iHNo-1) * rTheta1) * rFSDsTemp(3,iHNo) + sin((iHNo-1) * rTheta1) * rFSDsTemp(4,iHNo);

rStarFSDs(4,iHNo) = -sin((iHNo-1) * rTheta1) * rFSDsTemp(3,iHNo) + cos((iHNo-1) * rTheta1)

- rFSDsTemp(4,iHNo);

end % for iHNo = 1 : iNoOfHarmonicsAnalyse

rPsi1 = atan(rStarFSDs(3,2) / rStarFSDs(1,2));

rSemiMajor = sqrt(rStarFSDs(1,2)^2 + rStarFSDs(3,2)^2); % find the semi-major axis of the first ellipse

rFSDs(:,:) = rStarFSDs(:,:) ./ rSemiMajor; % if we haven't asked for normalisation of orientation, % return the coefficients normalised for starting point and size if bNormaliseOrientationState == 1

% now find the orientation normalised values - return them in rFSDs for iHNo = 1 : iNoOfHarmonicsAnalyse

rFSDsTemp(1,iHNo) = (cos(rPsi1) * rStarFSDs(1,iHNo) + sin(rPsi1) * rStarFSDs(3,iHNo)) / rSemiMajor;

rFSDsTemp(2,iHNo) = (cos(rPsi1) * rStarFSDs(2,iHNo) + sin(rPsi1) * rStarFSDs(4,iHNo)) / rSemiMajor;

rFSDsTemp(3,iHNo) = (-sin(rPsi1) * rStarFSDs(1,iHNo) + cos(rPsi1) * rStarFSDs(3,iHNo)) / rSemiMajor;

rFSDsTemp(4,iHNo) = (-sin(rPsi1) * rStarFSDs(2,iHNo) + cos(rPsi1) * rStarFSDs(4,iHNo)) / rSemiMajor;

end % for iHNo = 1 : iNoOfHarmonicsAnalyse rFSDs = rFSDsTemp; % return fully normlised coefficients end

end % if (bNormaliseSizeState == 1) || (bNormaliseOrientationState == 1)

return

function outln = rEfourier( rFSDs, iNoOfHarmonicsReconstruct,iNoOfPointsReconstruct)

% Reverse elliptical Fourier transform on the input Fourier series

% rFSDs generated by fEfourier.m. This reconstructs an approximation to the original outline figure

% using the specified number of harmonics and data points. See Kuhl FP and Giardina CR % "Elliptic Fourier features of a closed contour" Computer Graphics and % Image Processing 18:236-258 1982 for theory and details.

iStartHarmonic = 2; % start at 2 - No.1 is just an offset and is added in later (lines 17 & 27)

ReconnedOutline = 0; % reconstruct the x-projection for iTime = 1:iNoOfPointsReconstruct

rSum = 0.0;

for iHNo = iStartHarmonic:iNoOfHarmonicsReconstruct rSum = rSum + (rFSDs(1,iHNo) * cos(2*(iHNo-1)*pi*iTime / iNoOfPointsReconstruct) + ...

rFSDs(2,iHNo) * sin(2*(iHNo-1)*pi*iTime / iNoOfPointsReconstruct));

end % for iHNo = 1 : iNoOfHarmonicsReconstruct ReconnedOutline(iTime,1) = rFSDs(1,1) + rSum; end % for iTime = 1 : iNoOfPointsReconstruct

% reconstruct the y-projection for iTime = 1:iNoOfPointsReconstruct

rSum = 0.0;

for iHNo = iStartHarmonic:iNoOfHarmonicsReconstruct rSum = rSum + (rFSDs(3,iHNo) * cos(2*(iHNo-1)*pi*iTime / iNoOfPointsReconstruct) + ...

rFSDs(4,iHNo) * sin(2*(iHNo-1)*pi*iTime / iNoOfPointsReconstruct));

end % for iHNo = 1 : iNoOfHarmonicsReconstruct ReconnedOutline(iTime,2) = rFSDs(3,1) + rSum; end % for iTime = 1 : iNoOfPointsReconstruct outln = ReconnedOutline;

return

function k_coeff = calc_k_coefficent(x, y)

x_diff = circular_diff(x, 1); x_diff2 = circular_diff(x_diff, 1); y_diff = circular_diff(y, 1);

y_diff2 = circular_diff(y_diff, 1);

k_coeff = zeros(1, length(x_diff)); for t = 1: length(x_diff)

numerator = x_diff(t) * y_diff2(t) - y_diff(t) * x_diff2(t); denominator = (x_diff(t)^2 + y_diff(t)^2)^(3/2); k_coeff(t) = numerator / denominator; end

return

% do a simple linear difference differentiation on circular data function yy = circular_diff(xx, interval) yy = xx; for i = 1: length(xx) if (i == 1)

yy(i) = (xx(i + 1) - xx(end)) / (2 * interval); continue end

if (i == length(xx))

yy(i) = (xx(1) - xx(i - 1)) / (2 * interval); continue end

yy(i) = (xx(i + 1) - xx(i - 1)) / (2 * interval); end return

### S10. MATLAB code for size standardisation of thickness maps

function standardise_gridto1

%This function will standardise a matrix so that all values fall between 0

%and 1. It can also be used to standardise values according to the matrices

%median values by uncommenting the appropriate commands

% first get a folder from the user

folder_name = uigetdir('', 'Choose the folder that contains the landmark coordinates');

if folder_name == 0

error('No folder selected');

end

% make a series of folders for your results to go into

file_list = dir(fullfile(folder_name,'*.csv'));

if isempty(file_list)

error('No .csv files found in folder')

end

if ~exist([folder_name '\normgrids0_1\'],'dir')

mkdir([folder_name '\normgrids0_1\'])

end

%if ~exist([folder_name '\normgridstomedian\'],'dir')

%mkdir([folder_name '\normgridstomedian\'])

%end

if ~exist([folder_name '\norm_heatmap0_1\'],'dir')

mkdir([folder_name '\norm_heatmap0_1\'])

end

if ~exist([folder_name '\norm_heatmap0_1b\'],'dir')

mkdir([folder_name '\norm_heatmap0_1b\'])

end

file_number = 1:length(file_list)

file_name = [folder_name '/' file_list(file_number).name];

[pathstr, name, ext] = fileparts(file_name);

%This standardises all the values to the overall median value of the matrix

for file_number = 1:length(file_list)

file_name = [folder_name '/' file_list(file_number).name];

grid = csvread(file_name);

ncols = size(grid,2)

grid2 = grid([1:120],[1:ncols])

gridmax = max(grid2(:))

gridmin = min(grid2(:))

standmin = (grid2-gridmin)

stan0_1 = standmin./(gridmax-gridmin)

%medianvalue = median(grid2(:))

%standardised_grid = 1./grid2

%standardised_grid2=grid2-medianvalue

heatmap_name = [folder_name '\norm_heatmap0_1\' file_list(file_number).name(1:end-4) 'stand_heatmap'];

heatmap_name2 = [folder_name '\norm_heatmap0_1b\' file_list(file_number).name(1:end-4) 'stand_heatmap2'];

csvwrite([folder_name '\normgrids0_1\' file_list(file_number).name(1:end-4) 'normgrid.csv'], stan0_1);

% csvwrite([folder_name '\normgridstomedian\' file_list(file_number).name(1:end-4) 'normgrid2.csv'], standardised_grid2);

graph_bone_name = [file_list(file_number).name(1:end-4)];

figure(1)

clf('reset');

set(gcf, 'Color', 'w');

set(gcf,'Units','pixels');

set(gcf, 'Position', [50, 50, 1000, 1000]);

imagesc(stan0_1)

%plot_name = heatmap_name

set(gcf,'PaperType','<custom>');

set(gcf,'Units','inches');

set(gcf,'PaperUnits','inches');

position = get(gcf,'Position');

set(gcf,'PaperPosition',[0,0,position(3:4)]);

set(gcf,'PaperSize',position(3:4));

%axes1 = axes('Parent',figure1,...

plot_name = [heatmap_name];

colormap(jet(256));

colorbar('EastOutside');

xlabel('Bone Position');

ylabel({'Slice number.','Lower numbers are more proximal'});

%MODIFY THIS TITLE AS SEEN FIT

title(['Standardised cortical thickness of ' file_list(file_number).name(1:13) ' humerus']);

print(gcf,'-dtiff',[plot_name, '.png'],'-r 72')

figure(2)

clf('reset');

set(gcf, 'Color', 'w');

set(gcf,'Units','pixels');

set(gcf, 'Position', [50, 50, 1000, 1000]);

imagesc(stan0_1)

set(gcf,'PaperType','<custom>');

set(gcf,'Units','inches');

set(gcf,'PaperUnits','inches');

position = get(gcf,'Position');

set(gcf,'PaperPosition',[0,0,position(3:4)]);

set(gcf,'PaperSize',position(3:4));

plot_name2 = [heatmap_name2];

colormap(jet(256));

print(gcf,'-dtiff',[plot_name2, '.png'],'-r 72')

end

### S11 MATLAB code for combining matrices and running PCA.

function combine_run_pca

% first get a folder from the user

folder_name = uigetdir('', 'Choose the folder that contains the landmark coordinates');

if folder_name == 0

error('No folder selected');

end

file_list = dir(fullfile(folder_name,'*.csv'));

if isempty(file_list)

error('No .csv files found in folder')

end

for file_number = 1:length(file_list)

file_name = [folder_name '/' file_list(file_number).name];

data1 = csvread(file_name);

data1 = data1(1:120,1:360)

data1a=reshape(data1,43200,1)

combineda(file_number,:) = horzcat(data1a);

end

%Runs PCA and exports csv files of scores.Replace *NAME* with an appropriate prefix.

[coeff,score,latent,tsquared,explained,mu] = pca(combineda)

csvwrite('*NAME*combinedvaluesforpca.csv',combineda)

csvwrite('*NAME*_scores.csv',score)

csvwrite('*NAME*_coeff.csv',coeff)

csvwrite('*NAME*_explained.csv',explained)

end
